# Supplementary material for: Instructions and experiential learning have similar impacts on pain and pain-related brain responses but produce dissociations in value-based reversal learning
Source: eLife. 2022 Nov 1;11:e73353. doi: 10.7554/eLife.73353 (PMC9681218; doi:10.7554/eLife.73353)
Supplement: Figure 2—source data 1. [file elife-73353-fig2-data1.docx]

Figure 2–Source Data 1. Heat intensity effects on heat-evoked autonomic responses.^a^

^a^. This table presents results of linear mixed models predicting autonomic responses (skin conductance response (SCR) and pupil dilation (PD)) as a function of Heat Level (High vs Medium vs Low), Group (Instructed vs Uninstructed), Cue (Original High vs Original Low), and Phase (Original vs Reversed). SCR analyses included 36 participants, while PD analyses included 20 participants. See Methods and Table 1 for additional information.

^b^. Estimates based on a linear mixed effects model implemented in the “lmer” function of lme4 (Bates et al., 2015) using the following code: lmer(DV~Group*Templevels*Cue*Phase+(1+Templevels+Cue*Phase||Subject)).

^c^. Estimates based on a linear mixed effects model implemented in the “lme” function of nlme (Pinheiro et al., 2021) including autoregression using the following code: lme(DV~Group*Templevels*Cue*Phase, random=~1+Templevels+Cue*Phase|Subject, correlation=corAR1(), na.action=na.exclude).

^d^. Estimates based on Bayesian model linear mixed models using the “brms” function (Bürkner, 2017) using the following code: brm(SCR~Group*Templevels*Cue*Phase+(1+Templevels+Cue*Phase|Subject,prior=set_prior("normal(0,2.5)", class="b"), save_all_pars=TRUE, silent=TRUE, refresh=0, iter = 4000, warmup = 1000). The Region of Partial Equivalence (ROPE) was defined as [-0.03, 0.03] for SCR, [-31.00, 31.00] for mean PD, and [-4321.68, 4321.68] for PD area under the curve.

Figure 2–Source Data 2. Heat intensity effects: Small-volumes corrected results.^b^

| **Correction** | **Analysis** | **Effect** | **Anatomical label** | **x** | **y** | **z** | **# of voxels** | **Volume (mm^3^)** |
| --- | --- | --- | --- | --- | --- | --- | --- | --- |
| Pain modulatory network | *Main effect, Controlling for Group* | *FDR = .028; see uncorrected instead* | |  |  |  |  |  |
|  | *Group differences (Instructed - Uninstructed)* | Positive effect | *no voxels survive* |  |  |  |  |  |
|  |  | Negative effect | *no voxels survive* |  |  |  |  |  |
| Whole brain correction | *Main effect, Controlling for Group* | *FDR = .012; see uncorrected instead* | |  |  |  |  |  |
|  | *Group differences (Instructed - Uninstructed)* | Positive effect | *no voxels survive* |  |  |  |  |  |
|  |  | Negative effect | L Hippocampus (CA1) | -32 | -10 | -22 | 29 | 783 |
|  |  |  | R Postcentral Gyrus ( Area 4a ) | 22 | -34 | 62 | 2 | 54 |

^b^. This table presents group results from voxelwise analyses of associations between heat intensity and brain activation, as measured by AUC estimates (see Methods). Group results were analyzed using robust regression. Top rows are FDR-corrected within pain modulatory networks (see Figure 4 – Figure Supplement 1) and bottom rows are whole-brain corrected. Both analyses are FDR-corrected at q < .05. See Methods for additional details.

Figure 2–Source Data 3. Heat intensity effects: Uncorrected results.^c^

| **Analysis** | **Effect** | **Anatomical label** | **x** | **y** | **z** | **# of voxels** | **Volume (mm^3^)** |
| --- | --- | --- | --- | --- | --- | --- | --- |
| *Main effect, Controlling for Group* | Positive effect | L Cerebelum, contiguous with midbrain and occipital cortex | -8 | -70 | -20 | 2519 | 68013 |
|  |  | Bilateral insula, contiguous with bilateral striatum, dorsal ACC, left amygdala | 2 | -2 | 10 | 8794 | 237438 |
|  |  | R Middle Temporal Gyrus | 50 | -38 | -2 | 39 | 1053 |
|  |  | L Middle Frontal Gyrus (DLPFC) | -34 | 46 | 22 | 236 | 6372 |
|  |  | R Middle Frontal Gyrus (DLPFC) | 28 | 46 | 26 | 203 | 5481 |
|  | Negative effect | L Cerebelum IX | -4 | -52 | -50 | 18 | 486 |
|  |  | Bilateral insula, contiguous with bilateral striatum, dorsal ACC, left amygdala | 2 | -2 | 10 | 8794 | 237438 |
|  |  | Occipital cortex | 40 | -58 | 2 | 1132 | 30564 |
|  |  | R Superior Temporal Gyrus ( Area TE 3 ) | 58 | -10 | -10 | 191 | 5157 |
|  |  | L Middle Temporal Gyrus ( Area TE 3 ) | -56 | -4 | -14 | 88 | 2376 |
|  |  | L Mid Orbital Gyrus ( Area s32 ) | -4 | 26 | -14 | 243 | 6561 |
|  |  | R Precuneus | 10 | -56 | 22 | 207 | 5589 |
|  |  | L Superior Temporal Gyrus | -58 | -44 | 14 | 63 | 1701 |
|  |  | R Postcentral Gyrus | 52 | -16 | 52 | 104 | 2808 |
|  |  | L Postcentral Gyrus ( Area 1 ) | -56 | -16 | 46 | 67 | 1809 |
|  |  | R Superior Frontal Gyrus | 22 | 26 | 52 | 87 | 2349 |
|  |  | L Paracentral Lobule ( Area 4a ) | -2 | -28 | 62 | 74 | 1998 |
| *Group differences (Instructed - Uninstructed)* | Positive effect | R Fusiform Gyrus | 34 | -2 | -34 | 10 | 270 |
|  |  | L Hippocampus (CA1) | -32 | -10 | -22 | 37 | 999 |
|  |  | R Postcentral Gyrus ( Area PFt (IPL)) | 50 | -22 | 34 | 15 | 405 |
|  | Negative effect | L Inferior Parietal Lobule ( Area hIP1 (IPS)) | -44 | -50 | 44 | 35 | 945 |

^c^. This table presents uncorrected results from uncorrected voxelwise analyses (p < .001) of associations between heat intensity and brain activation, as measured by AUC estimates (see Methods). Group results were analyzed using robust regression.
