# Supplementary material for: Instructions and experiential learning have similar impacts on pain and pain-related brain responses but produce dissociations in value-based reversal learning
Source: eLife. 2022 Nov 1;11:e73353. doi: 10.7554/eLife.73353 (PMC9681218; doi:10.7554/eLife.73353)
Supplement: Figure 2—source data 2. [file elife-73353-fig2-data2.docx]

Figure 2–Source Data 3. Heat intensity effects: Uncorrected results.^c^

| **Analysis** | **Effect** | **Anatomical label** | **x** | **y** | **z** | **# of voxels** | **Volume (mm^3^)** |
| --- | --- | --- | --- | --- | --- | --- | --- |
| *Main effect, Controlling for Group* | Positive effect | L Cerebelum, contiguous with midbrain and occipital cortex | -8 | -70 | -20 | 2519 | 68013 |
|  |  | Bilateral insula, contiguous with bilateral striatum, dorsal ACC, left amygdala | 2 | -2 | 10 | 8794 | 237438 |
|  |  | R Middle Temporal Gyrus | 50 | -38 | -2 | 39 | 1053 |
|  |  | L Middle Frontal Gyrus (DLPFC) | -34 | 46 | 22 | 236 | 6372 |
|  |  | R Middle Frontal Gyrus (DLPFC) | 28 | 46 | 26 | 203 | 5481 |
|  | Negative effect | L Cerebelum IX | -4 | -52 | -50 | 18 | 486 |
|  |  | Bilateral insula, contiguous with bilateral striatum, dorsal ACC, left amygdala | 2 | -2 | 10 | 8794 | 237438 |
|  |  | Occipital cortex | 40 | -58 | 2 | 1132 | 30564 |
|  |  | R Superior Temporal Gyrus ( Area TE 3 ) | 58 | -10 | -10 | 191 | 5157 |
|  |  | L Middle Temporal Gyrus ( Area TE 3 ) | -56 | -4 | -14 | 88 | 2376 |
|  |  | L Mid Orbital Gyrus ( Area s32 ) | -4 | 26 | -14 | 243 | 6561 |
|  |  | R Precuneus | 10 | -56 | 22 | 207 | 5589 |
|  |  | L Superior Temporal Gyrus | -58 | -44 | 14 | 63 | 1701 |
|  |  | R Postcentral Gyrus | 52 | -16 | 52 | 104 | 2808 |
|  |  | L Postcentral Gyrus ( Area 1 ) | -56 | -16 | 46 | 67 | 1809 |
|  |  | R Superior Frontal Gyrus | 22 | 26 | 52 | 87 | 2349 |
|  |  | L Paracentral Lobule ( Area 4a ) | -2 | -28 | 62 | 74 | 1998 |
| *Group differences (Instructed - Uninstructed)* | Positive effect | R Fusiform Gyrus | 34 | -2 | -34 | 10 | 270 |
|  |  | L Hippocampus (CA1) | -32 | -10 | -22 | 37 | 999 |
|  |  | R Postcentral Gyrus ( Area PFt (IPL)) | 50 | -22 | 34 | 15 | 405 |
|  | Negative effect | L Inferior Parietal Lobule ( Area hIP1 (IPS)) | -44 | -50 | 44 | 35 | 945 |

^c^. This table presents uncorrected results from uncorrected voxelwise analyses (p < .001) of associations between heat intensity and brain activation, as measured by AUC estimates (see Methods). Group results were analyzed using robust regression.
