# Supplementary material for: Instructions and experiential learning have similar impacts on pain and pain-related brain responses but produce dissociations in value-based reversal learning
Source: eLife. 2022 Nov 1;11:e73353. doi: 10.7554/eLife.73353 (PMC9681218; doi:10.7554/eLife.73353)
Supplement: Figure 4—source data 1. [file elife-73353-fig4-data1.docx]

Figure 4–Source Data 1. Cue effects on skin conductance responses.^a^

^a^. This table presents results of linear mixed models predicting skin conductance response (SCR) as a function of Group (Instructed vs Uninstructed), Cue (Original High vs Original Low), and Phase (Original vs Reversed). Heat-evoked SCR analyses included 30 participants amd were limited to medium heat, while cue-evoked SCR included 29 participants and included all temperatures. See Methods and Table 1 for additional information.

^b^. Estimates based on a linear mixed effects model implemented in the “lmer” function of lme4 (Bates et al., 2015) using the following code: lmer(DV~Group*Templevels*Cue*Phase+(1+Templevels+Cue*Phase||Subject)).

^c^. Estimates based on a linear mixed effects model implemented in the “lme” function of nlme (Pinheiro et al., 2021) including autoregression using the following code: lme(DV~Group*Templevels*Cue*Phase, random=~1+Templevels+Cue*Phase|Subject, correlation=corAR1(), na.action=na.exclude).

^d^. Estimates based on Bayesian model linear mixed models using the “brms” function (Bürkner, 2017) using the following code: brm(SCR~Group*Templevels*Cue*Phase+(1+Templevels+Cue*Phase|Subject,prior=set_prior("normal(0,2.5)", class="b"), save_all_pars=TRUE, silent=TRUE, refresh=0, iter = 4000, warmup = 1000). The Region of Partial Equivalence (ROPE) was defined as [-0.03, 0.03] for SCR, [-31.00, 31.00] for mean PD, and [-4321.68, 4321.68] for PD area under the curve.
