# Supplementary material for: Instructions and experiential learning have similar impacts on pain and pain-related brain responses but produce dissociations in value-based reversal learning
Source: eLife. 2022 Nov 1;11:e73353. doi: 10.7554/eLife.73353 (PMC9681218; doi:10.7554/eLife.73353)
Supplement: Figure 5—source data 2. [file elife-73353-fig5-data2.docx]

Figure 5–Source Data 2. Mediation of current cue contingencies: Uncorrected results.^b^

| **Analysis** | **Effect** | **Anatomical label** | **x** | **y** | **z** | **# of voxels** | **Volume (mm^3^)** |
| --- | --- | --- | --- | --- | --- | --- | --- |
| All participants, regardless of Group | Path a, positive | R Cerebelum VIII | 22 | -62 | -50 | 44 | 1188 |
|  |  | L Cerebellum | -34 | -52 | -40 | 9 | 243 |
|  |  | Area hOc3v [V3v] | 28 | -94 | -20 | 22 | 594 |
|  |  | R Insula Lobe | 40 | 26 | -4 | 119 | 3213 |
|  |  | R Superior Orbital Gyrus ( Area Fp1 ) | 16 | 58 | -8 | 27 | 729 |
|  |  | L Insula Lobe | -46 | 8 | 2 | 134 | 3618 |
|  |  | L Caudate Nucleus | -14 | 16 | 2 | 31 | 837 |
|  |  | R Caudate Nucleus | 14 | 16 | 2 | 41 | 1107 |
|  |  | R IFG p. Triangularis ( Area 45 ) | 46 | 28 | 10 | 30 | 810 |
|  |  | R IFG p. Opercularis ( Area 45 ) | 58 | 16 | 20 | 34 | 918 |
|  |  | L Precentral Gyrus ( Area 4p ) | -34 | -20 | 50 | 176 | 4752 |
|  |  | R SupraMarginal Gyrus ( Area PFm (IPL)) | 62 | -46 | 32 | 57 | 1539 |
|  |  | L Posterior-Medial Frontal (dACC) | -8 | -16 | 50 | 183 | 4941 |
|  |  | L Precentral Gyrus | -46 | -2 | 28 | 10 | 270 |
|  |  | R Middle Cingulate Cortex | 8 | -4 | 32 | 11 | 297 |
|  |  | R Middle Frontal Gyrus (DLPFC) | 44 | 44 | 32 | 6 | 162 |
|  |  | R Superior Frontal Gyrus (DMPFC) | 14 | 26 | 50 | 121 | 3267 |
|  |  | RPrecentral Gyrus | 28 | -8 | 56 | 52 | 1404 |
|  |  | R Superior Frontal Gyrus (DMPFC) | 20 | -2 | 70 | 12 | 324 |
|  | Path a, negative | L Rectal Gyrus ( Area Fo2 , sgACC ) | -8 | 16 | -20 | 19 | 513 |
|  |  | L Superior Orbital Gyrus ( Area Fo2 , mOFC / sgACC ) | -14 | 28 | -20 | 10 | 270 |
|  |  | R Hippocampus (CA2) | 32 | -38 | -2 | 47 | 1269 |
|  |  | L Calcarine Gyrus | -10 | -56 | 8 | 19 | 513 |
|  | Path b, positive | L Cerebelum VIII | -32 | -56 | -50 | 157 | 4239 |
|  |  | R Cerebelum VIII | 32 | -62 | -52 | 31 | 837 |
|  |  | L Cerebelum Crus 2 | -28 | -76 | -38 | 53 | 1431 |
|  |  | R Cerebelum VI | 32 | -56 | -32 | 34 | 918 |
|  |  | L Cerebelum Crus 1 | -40 | -58 | -28 | 73 | 1971 |
|  |  | L Temporal Pole | -40 | 4 | -16 | 90 | 2430 |
|  |  | R Insula Lobe, contiguous with putamen | 38 | 10 | 8 | 1064 | 28728 |
|  |  | L Calcarine Gyrus ( Area hOc1 [V1]) | -4 | -76 | 10 | 731 | 19737 |
|  |  | L lateral hypothalamus | -4 | -2 | -8 | 68 | 1836 |
|  |  | R VLPFC / lateral OFC | 22 | 46 | -10 | 46 | 1242 |
|  |  | L Putamen | -22 | 14 | -2 | 48 | 1296 |
|  |  | L Rolandic Operculum | -58 | 4 | 4 | 26 | 702 |
|  |  | R Calcarine Gyrus ( Area hOc2 [V2]) | 26 | -100 | 2 | 25 | 675 |
|  |  | L DLPFC | -32 | 28 | 16 | 55 | 1485 |
|  |  | L Thalamus | -28 | -20 | 10 | 19 | 513 |
|  |  | L Insula Lobe | -34 | 10 | 16 | 22 | 594 |
|  |  | L IFG p. Opercularis ( Area 44 ) | -62 | 10 | 20 | 6 | 162 |
|  |  | L Superior Parietal Lobule | -20 | -64 | 40 | 26 | 702 |
|  |  | L rACC | -14 | 20 | 40 | 27 | 729 |
|  |  | L Superior Frontal Gyrus | -16 | 4 | 50 | 6 | 162 |
|  |  | L Precuneus ( Area 7P (SPL)) | -10 | -76 | 56 | 18 | 486 |
|  |  | L Postcentral Gyrus ( Area 4a ) | -20 | -28 | 62 | 55 | 1485 |
|  |  | RPrecentral Gyrus | 20 | -26 | 68 | 97 | 2619 |
|  |  | R Superior Parietal Lobule ( Area 7PC (SPL)) | 26 | -50 | 62 | 21 | 567 |
|  |  | L Postcentral Gyrus | -16 | -40 | 76 | 4 | 108 |
|  | Path b, Negative | L Inferior Temporal Gyrus | -56 | -22 | -22 | 41 | 1107 |
|  |  | L Hippocampus (DG) | -28 | -20 | -20 | 29 | 783 |
|  |  | L Mid Orbital Gyrus ( Area s32 ) | -8 | 32 | -14 | 15 | 405 |
|  |  | L Superior Medial Gyrus ( Area Fp2 ) | -2 | 64 | 2 | 78 | 2106 |
|  |  | R Middle Cingulate Cortex | 2 | -46 | 32 | 306 | 8262 |
|  |  | R Middle Temporal Gyrus | 46 | -62 | 20 | 83 | 2241 |
|  |  | L Angular Gyrus ( Area PGp (IPL)) | -46 | -70 | 32 | 29 | 783 |
|  | Path ab, Positive | R Middle Cingulate Cortex | 8 | -20 | 28 | 9 | 243 |
|  | Path ab, Negative | R Cerebelum VI | 20 | -58 | -28 | 28 | 756 |
|  |  | L Cerebelum VI | -28 | -50 | -22 | 23 | 621 |
|  |  | R Fusiform Gyrus ( Area FG4 ) | 40 | -44 | -22 | 19 | 513 |
|  |  | R Inferior Temporal Gyrus | 62 | -34 | -22 | 46 | 1242 |
|  |  | R Temporal Pole / Entorhinal cortex | 22 | 8 | -20 | 5 | 135 |
|  |  | R Mid Orbital Gyrus ( Area Fp2 ) | 10 | 52 | -10 | 16 | 432 |
|  |  | R Superior Temporal Gyrus ( Area TE 3 ) | 62 | 4 | -14 | 3 | 81 |
|  |  | R Superior Temporal Gyrus | 56 | -14 | -8 | 18 | 486 |
|  |  | L Middle Orbital Gyrus | -40 | 56 | -10 | 4 | 108 |
|  |  | L Operculum | -34 | -26 | -2 | 8 | 216 |
|  |  | L Calcarine Gyrus | -14 | -58 | 10 | 21 | 567 |
|  |  | L Middle Occipital Gyrus | -22 | -80 | 20 | 18 | 486 |
|  |  | R Superior Temporal Gyrus ( Area PGa (IPL)) | 58 | -50 | 20 | 39 | 1053 |
|  |  | L Superior Medial Gyrus | -4 | 52 | 26 | 30 | 810 |
|  |  | L SupraMarginal Gyrus ( Area PF (IPL)) | -64 | -38 | 28 | 16 | 432 |
|  |  | R Superior Occipital Gyrus | 28 | -80 | 38 | 19 | 513 |
|  |  | L Superior Medial Gyrus | 2 | 44 | 38 | 13 | 351 |
|  |  | L SupraMarginal Gyrus ( Area PF (IPL)) | -62 | -32 | 40 | 8 | 216 |
|  |  | R SupraMarginal Gyrus ( Area PF (IPL)) | 62 | -32 | 44 | 15 | 405 |
|  |  | R Inferior Parietal Lobule ( Area hIP2 (IPS)) | 44 | -44 | 52 | 9 | 243 |
|  |  | L Superior Frontal Gyrus | -14 | 14 | 58 | 33 | 891 |
| Moderation analysis, controlling for Group | Path a, positive | L IFG p. Opercularis | -46 | 10 | 2 | 74 | 1998 |
|  |  | L Middle Cingulate Cortex | -10 | -22 | 44 | 22 | 594 |
|  |  | L S1 | -28 | -14 | 46 | 30 | 810 |
|  |  | L Posterior-Medial Frontal | -10 | -8 | 58 | 47 | 1269 |
|  | Path a, negative | L Rectal Gyrus ( Area Fo2 ) | -8 | 14 | -20 | 11 | 297 |
|  |  | R Hippocampus | 34 | -38 | -2 | 24 | 648 |
|  | Path b, positive | L Cerebelum VIII | -32 | -52 | -50 | 82 | 2214 |
|  |  | L Cerebelum Crus 2 | -32 | -76 | -38 | 33 | 891 |
|  |  | L Cerebelum Crus 1 | -40 | -58 | -28 | 50 | 1350 |
|  |  | L Temporal Pole | -40 | 8 | -20 | 28 | 756 |
|  |  | R Insula Lobe | 40 | 14 | 2 | 246 | 6642 |
|  |  | L Lingual Gyrus ( Area hOc3v [V3v]) | -14 | -68 | -2 | 114 | 3078 |
|  |  | R Putamen | 26 | 10 | -8 | 59 | 1593 |
|  |  | R VLPFC | 22 | 46 | -10 | 27 | 729 |
|  |  | L Putamen | -22 | 14 | 2 | 21 | 567 |
|  |  | R Caudate Nucleus | 16 | 14 | 16 | 71 | 1917 |
|  |  | R Insula Lobe | 34 | -14 | 22 | 100 | 2700 |
|  |  | L Caudate Nucleus | -20 | 16 | 20 | 15 | 405 |
|  |  | L rdACC | -14 | 20 | 40 | 15 | 405 |
|  | Path b, Negative | L Hippocampus (DG) | -28 | -20 | -20 | 22 | 594 |
|  |  | R Precuneus | 2 | -52 | 20 | 63 | 1701 |
|  |  | R Middle Cingulate Cortex | 2 | -38 | 38 | 93 | 2511 |
|  |  | L Middle Cingulate Cortex | -14 | -50 | 38 | 20 | 540 |
|  | Path ab, Positive | *no voxels survive* |  |  |  |  |  |
|  | Path ab, Negative | *no voxels survive* |  |  |  |  |  |
| Group moderation of Path effects | Group x Path a, positive | L Posterior hippocampus | -34 | -40 | -4 | 58 | 1566 |
|  |  | L Caudate Nucleus | -14 | 4 | 20 | 117 | 3159 |
|  |  | R Precuneus | 10 | -56 | 26 | 25 | 675 |
|  | Group x Path a, negative | *no voxels survive* |  |  |  |  |  |
|  | Group x Path b, Positive | *no voxels survive* |  |  |  |  |  |
|  | Group x Path b, Negative | R Lingual Gyrus ( Area hOc2 [V2]) | 10 | -68 | -2 | 47 | 1269 |
|  | Group x Path ab, Positive | *no voxels survive* |  |  |  |  |  |
|  | Group x Path ab, Negative | *no voxels survive* |  |  |  |  |  |

^b^. This table presents uncorrected results from two voxelwise multilevel mediation analyses evaluating the effects of cues on subjective pain based on current contingencies (i.e. including reversals). All clusters are identified at a voxel-wise p-value of p < .001 (3 voxels at lowest threshold), contiguous with voxels at .005 and .01. Top rows report uncorrected results of each path across all participants, regardless of Group. Middle rows report Path a effects while controlling for Group. Bottom rows report clusters that are significantly moderated by Group. See Methods for additional details.
