# Supplementary material for: Instructions and experiential learning have similar impacts on pain and pain-related brain responses but produce dissociations in value-based reversal learning
Source: eLife. 2022 Nov 1;11:e73353. doi: 10.7554/eLife.73353 (PMC9681218; doi:10.7554/eLife.73353)
Supplement: Figure 6—source data 2. [file elife-73353-fig6-data2.docx]

Figure 6–Source Data 2. Mediation of original cue contingencies: Uncorrected results.^b^

| **Analysis** | **Effect** | **Anatomical label** | **x** | **y** | **z** | **# of voxels** | **Volume (mm^3^)** |
| --- | --- | --- | --- | --- | --- | --- | --- |
| All participants, regardless of Group | Path a, positive | Brainstem (RVM) | 4 | -38 | -40 | 48 | 1296 |
|  |  | L Fusiform Gyrus | -26 | -2 | -46 | 7 | 189 |
|  |  | Cerebellar Vermis 10 | 4 | -50 | -26 | 26 | 702 |
|  |  | Brainstem (Pons) | 4 | -22 | -22 | 17 | 459 |
|  |  | Area Fo3 (L OFC) | -20 | 50 | -20 | 8 | 216 |
|  |  | R Cuneus | 20 | -86 | 8 | 17 | 459 |
|  |  | Cerebellar Vermis 4/5 | 2 | -52 | 2 | 10 | 270 |
|  |  | R Thalamus (Prefrontal) | 10 | -14 | 8 | 66 | 1782 |
|  |  | R Middle Frontal Gyrus (DLPFC) | 38 | 62 | 8 | 39 | 1053 |
|  |  | R Middle Occipital Gyrus ( Area hOc4la) | 38 | -80 | 8 | 25 | 675 |
|  |  | R Middle Frontal Gyrus (DLPFC) | 50 | 26 | 38 | 49 | 1323 |
|  |  | R Middle Cingulate Cortex | 8 | -22 | 32 | 19 | 513 |
|  |  | L Superior Parietal Lobule | -14 | -74 | 40 | 17 | 459 |
|  |  | R Precuneus ( Area 7A (SPL)) | 4 | -68 | 62 | 61 | 1647 |
|  |  | R Superior Parietal Lobule ( Area 7A (SPL)) | 32 | -58 | 62 | 11 | 297 |
|  | Path a, negative | R Hippocampus (CA2) | 26 | -16 | -16 | 48 | 1296 |
|  |  | R Precuneus | 14 | -40 | 2 | 26 | 702 |
|  |  | L Superior Temporal Gyrus ( Area TE 3 ) | -64 | -20 | 4 | 34 | 918 |
|  |  | R Posterior Hippocampus | 28 | -44 | 4 | 21 | 567 |
|  |  | R Precuneus | 14 | -56 | 16 | 33 | 891 |
|  |  | L Precuneus | -2 | -62 | 26 | 8 | 216 |
|  | Path b, positive | L Cerebelum VIII | -32 | -56 | -50 | 163 | 4401 |
|  |  | L Cerebelum Crus 2 | -28 | -80 | -38 | 49 | 1323 |
|  |  | R Cerebelum VI | 32 | -56 | -32 | 31 | 837 |
|  |  | L Cerebelum Crus 1 | -40 | -58 | -32 | 81 | 2187 |
|  |  | L Temporal Pole | -40 | 4 | -16 | 83 | 2241 |
|  |  | R Insula Lobe, contiguous with R striatum | 38 | 8 | 8 | 1176 | 31752 |
|  |  | L Calcarine Gyrus ( Area hOc2 [V2]) | -4 | -74 | 14 | 693 | 18711 |
|  |  | Bilateral hypothalamus, contiguous with L amygdala | -4 | -4 | -8 | 76 | 2052 |
|  |  | R VLPFC | 22 | 46 | -10 | 44 | 1188 |
|  |  | Area hOc1 [V1] | -14 | -106 | -2 | 24 | 648 |
|  |  | L Putamen | -22 | 14 | 2 | 53 | 1431 |
|  |  | L Rolandic Operculum | -58 | 4 | 4 | 28 | 756 |
|  |  | R Calcarine Gyrus ( Area hOc1 [V1]) | 22 | -100 | 2 | 25 | 675 |
|  |  | L Caudate Nucleus | -20 | 14 | 16 | 105 | 2835 |
|  |  | L Insula Lobe ( Area OP2 [PIVC]) | -34 | -20 | 16 | 45 | 1215 |
|  |  | L Middle Insula | -34 | 8 | 20 | 17 | 459 |
|  |  | R rdACC | 14 | 26 | 38 | 17 | 459 |
|  |  | L rdACC | -14 | 20 | 40 | 25 | 675 |
|  |  | L Superior Parietal Lobule ( Area 5L (SPL)) | -26 | -46 | 50 | 22 | 594 |
|  |  | L Postcentral Gyrus ( Area 4a ) | -20 | -28 | 62 | 52 | 1404 |
|  |  | L Precuneus ( Area 7P (SPL)) | -10 | -76 | 56 | 16 | 432 |
|  |  | L Precuneus ( Area 5M (SPL)) | -14 | -44 | 58 | 22 | 594 |
|  |  | R Postcentral Gyrus ( Area 4a ) | 22 | -34 | 64 | 144 | 3888 |
|  | Path b, negative | L Middle Temporal Gyrus | -56 | -20 | -22 | 28 | 756 |
|  |  | L Hippocampus (DG) | -28 | -20 | -20 | 29 | 783 |
|  |  | L Superior Medial Gyrus ( Area Fp1 ) | -2 | 68 | 8 | 49 | 1323 |
|  |  | L Occipital cortex | -58 | -70 | 16 | 7 | 189 |
|  |  | R Middle Temporal Gyrus | 46 | -64 | 20 | 72 | 1944 |
|  |  | R Middle Cingulate Cortex | 2 | -44 | 32 | 198 | 5346 |
|  |  | L Angular Gyrus ( Area PGp (IPL)) | -46 | -70 | 32 | 21 | 567 |
|  |  | L Middle Cingulate Cortex | -14 | -50 | 38 | 32 | 864 |
|  | Path ab, positive | *no voxels survive* |  |  |  |  |  |
|  | Path ab, negative | Lobule VI Hem | -22 | -58 | -32 | 9 | 243 |
|  |  | Cerebellar Vermis 6 | 4 | -64 | -22 | 8 | 216 |
|  |  | R Inferior Temporal Gyrus ( Area FG4 ) | 50 | -56 | -22 | 21 | 567 |
|  |  | R Cerebelum VI | 16 | -64 | -14 | 26 | 702 |
|  |  | L Mid Orbital Gyrus ( Area Fp1 ) | -4 | 62 | -14 | 8 | 216 |
|  |  | R Superior Temporal Gyrus | 52 | -10 | -10 | 9 | 243 |
|  |  | R Superior Temporal Gyrus ( Area TE 1.0) | 56 | -8 | 2 | 10 | 270 |
|  |  | R Superior Medial Gyrus ( Area Fp2 ) | 14 | 62 | 4 | 22 | 594 |
|  |  | L Cuneus ( Area hOc3d [V3d]) | -2 | -80 | 22 | 209 | 5643 |
|  |  | R Middle Temporal Gyrus ( Area hOc4la) | 50 | -70 | 14 | 23 | 621 |
|  |  | R Middle Occipital Gyrus | 34 | -82 | 14 | 29 | 783 |
|  |  | R Angular Gyrus | 46 | -46 | 26 | 49 | 1323 |
|  |  | L Precuneus | 2 | -58 | 26 | 11 | 297 |
|  |  | L Precentral Gyrus | -50 | -2 | 50 | 15 | 405 |
|  |  | R Inferior Parietal Lobule ( Area PFm (IPL)) | 56 | -44 | 52 | 11 | 297 |
|  |  | R Postcentral Gyrus ( Area 1 ) | 56 | -26 | 52 | 14 | 378 |
|  |  | RPrecentral Gyrus | 38 | -20 | 70 | 16 | 432 |
|  |  | L Precuneus ( Area 5L (SPL)) | -14 | -52 | 76 | 10 | 270 |
| Group moderation of Path effects | Path a, positive | Brainstem (RVM) | 4 | -38 | -40 | 29 | 783 |
|  |  | Brainstem (pons) | 2 | -22 | -22 | 12 | 324 |
|  | Path a, negative | R Hippocampus (CA2) | 28 | -16 | -16 | 31 | 837 |
|  |  | R Precuneus | 14 | -52 | 16 | 30 | 810 |
|  | Path b, positive | L Cerebelum VIII | -32 | -52 | -50 | 91 | 2457 |
|  |  | L Cerebelum Crus 2 | -32 | -76 | -38 | 35 | 945 |
|  |  | L Cerebelum Crus 1 | -40 | -58 | -28 | 49 | 1323 |
|  |  | L Anterior Insula, operculum | -40 | 8 | -16 | 37 | 999 |
|  |  | R Insula Lobe | 44 | 10 | -10 | 80 | 2160 |
|  |  | L Lingual Gyrus ( Area hOc3v [V3v]) | -14 | -68 | -2 | 130 | 3510 |
|  |  | Bilateral hypothalamus, contiguous with L amygdala | -4 | -2 | -8 | 44 | 1188 |
|  |  | R Putamen, contiguous with R Caudate, anterior insula | 26 | 10 | 8 | 324 | 8748 |
|  |  | R Lateral OFC | 22 | 50 | -10 | 20 | 540 |
|  |  | L Putamen | -22 | 14 | 2 | 25 | 675 |
|  |  | R Middle Insula | 34 | -10 | 26 | 138 | 3726 |
|  | Path b, negative | L Hippocampus (DG) | -28 | -20 | -20 | 19 | 513 |
|  |  | Cuneus | 2 | -52 | 16 | 60 | 1620 |
|  |  | R Middle Cingulate Cortex | 2 | -38 | 40 | 58 | 1566 |
|  |  | L Middle Cingulate Cortex | -14 | -50 | 38 | 16 | 432 |
|  | Path ab, positive | *No voxels survive* |  |  |  |  |  |
|  | Path ab, negative | *No voxels survive* |  |  |  |  |  |
|  | Group x Path a, positive | R Rectal Gyrus ( Area Fo1, medial OFC ) | 14 | 32 | -22 | 12 | 324 |
|  | Group x Path a, negative | *No voxels survive* |  |  |  |  |  |
|  | Group x Path b, positive | *No voxels survive* |  |  |  |  |  |
|  | Group x Path b, negative | R Lingual Gyrus ( Area hOc2 [V2]) | 10 | -68 | -2 | 46 | 1242 |
|  | Group x Path ab, positive | *No voxels survive* |  |  |  |  |  |
|  | Group x Path ab, negative | *No voxels survive* |  |  |  |  |  |

^b^. This table presents uncorrected results from two voxelwise multilevel mediation analyses evaluating the effects of cues on subjective pain based on original contingencies while controlling for reversals. All clusters are identified at a voxel-wise p-value of p < .001 (3 voxels at lowest threshold), contiguous with voxels at .005 and .01. Top rows report uncorrected results of each path across all participants, regardless of Group. Middle rows report Path a effects while controlling for Group. Bottom rows report clusters that are significantly moderated by Group. See Methods for additional details.
