# Supplementary material for: Instructions and experiential learning have similar impacts on pain and pain-related brain responses but produce dissociations in value-based reversal learning
Source: eLife. 2022 Nov 1;11:e73353. doi: 10.7554/eLife.73353 (PMC9681218; doi:10.7554/eLife.73353)
Supplement: Figure 8—source data 1. [file elife-73353-fig8-data1.docx]

Figure 6–Source Data 1. Associations with expected value (EV): Small-volumes corrected results.^a^

| **Correction** | **Analysis** | **Effect** | **Anatomical label** | **x** | **y** | **z** | **# of voxels** | **Volume (mm^3^)** |
| --- | --- | --- | --- | --- | --- | --- | --- | --- |
| Pain modulatory network | Instructed Group | Positive association with EV | *No voxels survive* | | | | | |
|  |  | Negative association with EV | *No voxels survive* | | | | | |
|  | Uninstructed Group | Positive association with EV | *No voxels survive* | | | | | |
|  |  | Negative association with EV | *No voxels survive* | | | | | |
|  | Main effect of EV, controlling for Group | Positive association with EV | *No voxels survive* | | | | | |
|  |  | Negative association with EV | *No voxels survive* | | | | | |
|  | Group differences in EV (Instructed - Uninstructed) | Positive effect | L Entorhinal cortex | -38 | 2 | -20 | 5 | 135 |
|  |  |  | L rACC | -4 | 40 | 4 | 132 | 3564 |
|  |  |  | L Thalamus (Prefrontal) | -16 | -20 | 14 | 42 | 1134 |
|  |  |  | L Heschls Gyrus ( Area Ig2 ) | -38 | -20 | 10 | 18 | 486 |
|  |  |  | R Thalamus (Temporal) | 16 | -28 | 14 | 2 | 54 |
|  |  |  | R Thalamus (Temporal) | 8 | -14 | 16 | 6 | 162 |
|  |  | Negative effect | *No voxels survive* |  |  |  |  |  |
| Whole brain correction | Instructed Group | Positive association with EV | R Superior Frontal Gyrus (MPFC) | 16 | 56 | 16 | 6 | 162 |
|  |  | Negative association with EV | *No voxels survive* | | | | | |
|  | Uninstructed Group | Positive association with EV | *No voxels survive* | | | | | |
|  |  | Negative association with EV | L rACC | -16 | 40 | 16 | 2 | 54 |
|  | Main effect of EV, controlling for Group | Positive association with EV | *No voxels survive* | | | | | |
|  |  | Negative association with EV | *No voxels survive* | | | | | |
|  | Group differences in EV (Instructed - Uninstructed) | Positive effect | L Thalamus (Prefrontal) | -16 | -16 | 16 | 47 | 1269 |
|  |  |  | R Thalamus (Parietal) | 20 | -28 | 14 | 3 | 81 |
|  |  |  | L rACC (Superior Medial Gyrus) | -10 | 40 | 26 | 18 | 486 |
|  |  |  | L Middle Cingulate Cortex | -8 | -22 | 32 | 7 | 189 |
|  |  | Negative effect | *No voxels survive* | | | | | |

^a^. This table presents group results from voxelwise analyses of associations between expected value (based on fits to pain) and brain activation on medium heat, as measured by AUC estimates (see Methods). Group results were analyzed using robust regression. Top rows are FDR-corrected within pain modulatory networks (see Figure 4 – Figure Supplement 1) and bottom rows are whole-brain corrected. Both analyses are FDR-corrected at q < .05. See Methods for additional details.

Figure 6–Source Data 2. Associations with expected value (EV): Uncorrected results.^b^

| **Analysis** | **Effect** | **Anatomical label** | **x** | **y** | **z** | **# of voxels** | **Volume (mm^3^)** |
| --- | --- | --- | --- | --- | --- | --- | --- |
| Instructed Group | Positive association with EV | L Entorhinal cortex, contiguous with L Putamen | -32 | 2 | -8 | 36 | 972 |
|  |  | L posterior Putamen | -32 | -10 | 8 | 8 | 216 |
|  |  | R ACC / MPFC | 14 | 40 | 14 | 5 | 135 |
|  |  | R Superior Frontal Gyrus (DMPFC) | 16 | 56 | 16 | 9 | 243 |
|  |  | rdACC (area 33) | 8 | 16 | 22 | 52 | 1404 |
|  |  | L rdACC | -14 | 28 | 26 | 16 | 432 |
|  |  | Posterior Cingulate Cortex | 2 | -38 | 26 | 31 | 837 |
|  |  | L M1, contiguous with S1, posterior cingulate cortex, precuneus | -20 | -22 | 40 | 207 | 5589 |
|  | Negative association with EV | L Superior Orbital Gyrus ( Area Fo3 ) | -22 | 44 | -14 | 22 | 594 |
|  |  | R Calcarine Gyrus ( Area hOc1 [V1]) | 16 | -56 | 8 | 40 | 1080 |
|  |  | L IFG p. Triangularis (DLPFC) | -46 | 28 | 22 | 65 | 1755 |
| Uninstructed Group | Positive association with EV | *No voxels survive* | | | | | |
|  | Negative association with EV | L Temporal Pole | -40 | 8 | -20 | 48 | 1296 |
|  |  | L IFG p. Orbitalis | -34 | 34 | -16 | 16 | 432 |
|  |  | R Hippocampus (DG) | 28 | -34 | -4 | 58 | 1566 |
|  |  | L Hippocampus | -34 | -38 | -4 | 20 | 540 |
|  |  | L Superior Orbital Gyrus ( Area Fp1 ) | -20 | 62 | -2 | 31 | 837 |
|  |  | L PCC | -2 | -46 | 14 | 161 | 4347 |
|  |  | L Caudate Tail, contiguous with L Thalamus, L Middle Insula | -26 | -22 | 20 | 71 | 1917 |
|  |  | L Insula Lobe | -26 | 26 | 10 | 12 | 324 |
| Main effect of EV, controlling for Group | Positive association with EV | R Cerebelum VIII | 26 | -68 | -56 | 27 | 729 |
|  |  | R Inferior Occipital Gyrus ( Area hOc3v [V3v]) | 34 | -92 | -10 | 21 | 567 |
|  |  | R SupraMarginal Gyrus ( Area PFm (IPL)) | 58 | -44 | 28 | 35 | 945 |
|  |  | L Middle Cingulate Cortex | -8 | -20 | 40 | 31 | 837 |
|  |  | L Postcentral Gyrus ( Area 3a ) | -32 | -34 | 46 | 10 | 270 |
|  |  | L Precentral Gyrus | -34 | -10 | 50 | 49 | 1323 |
|  |  | L Posterior-Medial Frontal | -10 | -8 | 58 | 43 | 1161 |
|  | Negative association with EV | L Temporal Pole | -40 | 10 | -22 | 34 | 918 |
|  |  | L Superior Orbital Gyrus ( Area Fo3 ) | -14 | 50 | -20 | 23 | 621 |
|  |  | R Temporal Pole | 38 | 8 | -22 | 11 | 297 |
|  |  | L Rectal Gyrus ( Area Fo2 ) | -8 | 20 | -22 | 16 | 432 |
|  |  | R Fusiform Gyrus | 38 | -26 | -20 | 4 | 108 |
|  |  | R Hippocampus (DG) | 26 | -32 | -8 | 96 | 2592 |
|  |  | L IFG p. Orbitalis | -34 | 40 | -16 | 37 | 999 |
|  |  | L Superior Orbital Gyrus (Fp1) | -28 | 62 | -2 | 31 | 837 |
|  |  | L Calcarine Gyrus | -8 | -50 | 4 | 46 | 1242 |
|  |  | R Calcarine Gyrus | 14 | -50 | 10 | 122 | 3294 |
|  |  | Thal: Temporal | -20 | -34 | 4 | 9 | 243 |
|  |  | Thal: Parietal | -20 | -22 | 16 | 34 | 918 |
| Group differences in EV (Instructed - Uninstructed) | Positive effect | R Cerebelum Crus 2 | 44 | -50 | -46 | 13 | 351 |
|  |  | L Anterior Insula / Operculum | -38 | 10 | -16 | 85 | 2295 |
|  |  | R Cerebelum VI | 40 | -62 | -22 | 9 | 243 |
|  |  | L MPFC, contiguous with rACC | -4 | 44 | 8 | 523 | 14121 |
|  |  | R Hippocampus | 32 | -32 | -4 | 32 | 864 |
|  |  | R Occipital cortex | 26 | -86 | 2 | 41 | 1107 |
|  |  | L Superior Temporal Gyrus | -56 | -14 | -2 | 9 | 243 |
|  |  | L Thalamus (Parietal) | -16 | -22 | 14 | 123 | 3321 |
|  |  | L Insula Lobe ( Area Ig2 ) | -38 | -16 | 10 | 35 | 945 |
|  |  | L PCC | -2 | -46 | 20 | 196 | 5292 |
|  | Negative effect | R Inferior Temporal Gyrus | 62 | -40 | -16 | 17 | 459 |
|  |  | L Inferior Temporal Gyrus | -64 | -46 | -14 | 13 | 351 |

^b^. This table presents group results from voxelwise analyses of associations between expected value (based on fits to pain) and brain activation on medium heat, as measured by AUC estimates (see Methods). Group results were analyzed using robust regression. All clusters are identified at a voxel-wise p-value of p < .001 (3 voxels at lowest threshold), contiguous with voxels at .005 and .01. See Methods for additional details.

Figure 6–Source Data 3. Associations with expected value (EV) based on fits to heat-evoked SCR^c^

| **Correction** | **Analysis** | **Effect** | **Anatomical label** | **x** | **y** | **z** | **# of voxels** | **Volume (mm^3^)** |
| --- | --- | --- | --- | --- | --- | --- | --- | --- |
| Pain modulatory network | Instructed Group | Positive association with EV | L Putamen | -16 | 16 | -10 | 1 | 27 |
|  |  |  | R Insula Lobe | 32 | 20 | -10 | 8 | 216 |
|  |  |  | L sgACC | -10 | 34 | -4 | 5 | 135 |
|  |  |  | L rACC | 2 | 34 | 10 | 5 | 135 |
|  |  |  | R rACC | 14 | 44 | 14 | 4 | 108 |
|  |  | Negative association with EV | *No voxels survive* | | | | | |
|  | Uninstructed Group | Positive association with EV | *No voxels survive* | | | | | |
|  |  | Negative association with EV | *No voxels survive* | | | | | |
|  | Main effect of EV, controlling for Group | Positive effect | *No voxels survive* | | | | | |
|  |  | Negative effect | *No voxels survive* | | | | | |
|  | Group differences in EV (Instructed - Uninstructed) | Positive effect | *No voxels survive* | | | | | |
|  |  | Negative effect | *No voxels survive* | | | | | |
| Whole brain correction | Instructed Group | Positive association with EV | R Anterior Insula | 32 | 20 | -10 | 9 | 243 |
|  |  | Negative association with EV | *No voxels survive* | | | | | |
|  | Uninstructed Group | Positive association with EV | R DLPFC | 44 | 8 | 26 | 1 | 27 |
|  |  | Negative association with EV | L Anterior Insula | -26 | 26 | 10 | 12 | 324 |
|  |  |  | L rdACC | -16 | 40 | 16 | 2 | 54 |
|  | Main effect of EV, controlling for Group | Positive effect | *No voxels survive* | | | | | |
|  |  | Negative effect | *No voxels survive* | | | | | |
|  | Group differences in EV (Instructed - Uninstructed) | Positive effect | L Superior Medial Gyrus | -10 | 40 | 26 | 8 | 216 |
|  |  | Negative effect | *No voxels survive* | | | | | |
| Uncorrected | Instructed Group | Positive association with EV | L Putamen | -32 | 2 | -8 | 52 | 1404 |
|  |  |  | R Anterior Insula | 32 | 20 | -8 | 17 | 459 |
|  |  |  | R Caudate Nucleus | 16 | 20 | 4 | 33 | 891 |
|  |  |  | R rdACC | 14 | 40 | 14 | 7 | 189 |
|  |  |  | R rdACC | 8 | 16 | 22 | 29 | 783 |
|  |  |  | Middle Cingulate Cortex | 2 | -22 | 34 | 49 | 1323 |
|  |  | Negative association with EV | L OFC | -26 | 44 | -14 | 17 | 459 |
|  |  |  | R Retrosplenial Cortex | 20 | -52 | 8 | 14 | 378 |
|  |  |  | R Occipital Cortex | 8 | -64 | 14 | 23 | 621 |
|  |  |  | L DLPFC | -44 | 26 | 22 | 60 | 1620 |
|  | Uninstructed Group | Positive association with EV | R DLPFC | 58 | 16 | 16 | 16 | 432 |
|  |  | Negative association with EV | L Inferior Frontal Gyrus | -40 | 8 | -20 | 56 | 1512 |
|  |  |  | R Fusiform Gyrus | 28 | -34 | -4 | 61 | 1647 |
|  |  |  | L Fusiform Gyrus | -34 | -38 | -4 | 29 | 783 |
|  |  |  | L Prefrontal cortex | -22 | 64 | -2 | 21 | 567 |
|  |  |  | L Posterior hippocampus | -14 | -34 | 4 | 23 | 621 |
|  |  |  | L Anterior Insula | -26 | 28 | 10 | 25 | 675 |
|  |  |  | R Retrosplenial Cortex | 14 | -56 | 20 | 40 | 1080 |
|  |  |  | L Thalamus | -20 | -22 | 16 | 24 | 648 |
|  |  |  | L DMPFC | -14 | 40 | 26 | 8 | 216 |
|  | Main effect of EV, controlling for Group | Positive effect | R Cerebelum VIII | 26 | -68 | -56 | 26 | 702 |
|  |  |  | L Inferior Temporal Gyrus | -62 | -34 | -26 | 7 | 189 |
|  |  |  | R Inferior Occipital Gyrus ( Area hOc3v [V3v]) | 34 | -92 | -8 | 26 | 702 |
|  |  |  | R Insula Lobe | 32 | 22 | -4 | 34 | 918 |
|  |  |  | R MCC | 2 | -20 | 38 | 19 | 513 |
|  |  |  | L Postcentral Gyrus ( Area 3a ) | -32 | -34 | 46 | 6 | 162 |
|  |  |  | L Precentral Gyrus | -34 | -10 | 52 | 59 | 1593 |
|  |  |  | L Posterior-Medial Frontal | -10 | -8 | 58 | 29 | 783 |
|  |  | Negative effect | R Cerebelum Crus 1 | 14 | -76 | -28 | 4 | 108 |
|  |  |  | L medial OFC (Area Fo3 ) | -10 | 50 | -26 | 11 | 297 |
|  |  |  | R ParaHippocampal Gyrus | 28 | -38 | -8 | 39 | 1053 |
|  |  |  | R Precuneus | 16 | -46 | 10 | 47 | 1269 |
|  | Group differences in EV (Instructed - Uninstructed) | Positive effect | L Anterior Insula, contiguous with L Putamen | -38 | 4 | -16 | 78 | 2106 |
|  |  |  | L MPFC, ACC | -4 | 46 | 4 | 396 | 10692 |
|  |  |  | R Occipital Cortex | 26 | -86 | 2 | 57 | 1539 |
|  |  |  | L Thalamus (prefrontal) | -14 | -16 | 16 | 26 | 702 |
|  |  | Negative effect | *No voxels survive* | | | | | |

^c^. This table presents group results from voxelwise analyses of associations between expected value (based on fits to heat-evoked SCR) and brain activation on medium heat, as measured by AUC estimates (see Methods). Group results were analyzed using robust regression. See Methods for additional details.

Figure 6–Source Data 4. Associations with expected value (EV) based on fits to cue-evoked SCR^d^

| **Correction** | **Analysis** | **Effect** | **Anatomical label** | **x** | **y** | **z** | **# of voxels** | **Volume (mm^3^)** |
| --- | --- | --- | --- | --- | --- | --- | --- | --- |
| Pain modulatory network | Instructed Group | Positive association with EV | L midbrain (substantia nigra) | -8 | -22 | -10 | 4 | 108 |
|  |  |  | L Thalamus (Parietal) | -22 | -22 | 16 | 1 | 27 |
|  |  | Negative association with EV | L Superior Orbital Gyrus / Area Fo1 | -14 | 44 | -22 | 10 | 270 |
|  |  |  | L IFG p. Triangularis | -44 | 34 | 14 | 2 | 54 |
|  |  |  | L IFG p. Triangularis | -44 | 22 | 22 | 17 | 459 |
|  | Uninstructed Group | Positive association with EV | *No voxels survive* | | | | | |
|  |  | Negative association with EV | *No voxels survive* | | | | | |
|  | Main effect of EV, controlling for Group | Positive effect | L midbrain (substantia nigra) | -8 | -20 | -10 | 2 | 54 |
|  |  | Negative effect | L IFG p. Triangularis | -40 | 34 | 14 | 7 | 189 |
|  | Group differences in EV (Instructed - Uninstructed) | Positive effect | *No voxels survive* | | | | | |
|  |  | Negative effect | *No voxels survive* | | | | | |
| Whole brain correction | Instructed Group | Positive association with EV | L Midbrain (Substantia Nigra) | -8 | -22 | -10 | 4 | 108 |
|  |  |  | dACC | 4 | 16 | 16 | 12 | 324 |
|  |  |  | L PreSMA | -20 | -32 | 44 | 1 | 27 |
|  |  | Negative association with EV | L Superior Orbital Gyrus / Fo1 | -14 | 44 | -20 | 18 | 486 |
|  |  |  | L IFG p. Triangularis | -38 | 44 | 4 | 4 | 108 |
|  |  |  | L IFG p. Triangularis | -46 | 22 | 26 | 150 | 4050 |
|  | Uninstructed Group | Positive association with EV | RPrecentral Gyrus | 46 | 2 | 46 | 10 | 270 |
|  |  |  | RPrecentral Gyrus | 32 | -22 | 52 | 1 | 27 |
|  |  |  | R Superior Frontal Gyrus | 26 | -8 | 64 | 12 | 324 |
|  |  | Negative association with EV | *No voxels survive* | | | | | |
|  | Main effect of EV, controlling for Group | Positive effect | *No voxels survive* | | | | | |
|  |  | Negative effect | *No voxels survive* | | | | | |
|  | Group differences in EV (Instructed - Uninstructed) | Positive effect | *No voxels survive* | | | | | |
|  |  | Negative effect | *No voxels survive* | | | | | |
| Uncorrected | Instructed Group | Positive association with EV | L Putamen, contiguous with Amygdala | -32 | 2 | -8 | 20 | 540 |
|  |  |  | R Middle Occipital Gyrus / Area hOc4la | 40 | -76 | 4 | 51 | 1377 |
|  |  |  | L Superior Frontal Gyrus | -22 | 38 | 50 | 6 | 162 |
|  |  | Negative association with EV | L Superior Orbital Gyrus / Area Fo1 | -14 | 44 | -20 | 25 | 675 |
|  |  |  | L IFG p. Triangularis | -46 | 22 | 26 | 201 | 5427 |
|  | Uninstructed Group | Positive association with EV | L DMPFC | -16 | -8 | 58 | 54 | 1458 |
|  |  |  | RPrecentral Gyrus | 46 | 2 | 46 | 14 | 378 |
|  |  |  | RPrecentral Gyrus | 28 | -4 | 50 | 29 | 783 |
|  |  |  | R Superior Frontal Gyrus | 28 | -8 | 64 | 22 | 594 |
|  |  | Negative association with EV | L Temporal Pole, contiguous with Insula | -40 | 8 | -20 | 53 | 1431 |
|  |  |  | R Hippocampus (DG) | 32 | -34 | -8 | 51 | 1377 |
|  |  |  | L Hippocampus (DG) | -34 | -34 | -8 | 31 | 837 |
|  |  |  | L Middle Orbital Gyrus | -32 | 40 | -8 | 6 | 162 |
|  |  |  | L ACC, MPFC | -2 | 40 | 4 | 41 | 1107 |
|  |  |  | R Calcarine Gyrus | 10 | -58 | 16 | 8 | 216 |
|  |  |  | R Caudate Nucleus | 20 | -22 | 22 | 16 | 432 |
|  |  |  | L Superior Medial Gyrus | -14 | 40 | 26 | 7 | 189 |
|  | Main effect of EV, controlling for Group | Positive effect | L Middle Cingulate Cortex, M1 | -16 | -14 | 46 | 66 | 1782 |
|  |  |  | L Inferior Parietal Lobule ( Area 1 ) | -52 | -26 | 50 | 23 | 621 |
|  |  | Negative effect | R VLPFC | 28 | 40 | -8 | 6 | 162 |
|  |  |  | L IFG p. Triangularis | -46 | 28 | 22 | 138 | 3726 |
|  | Group differences in EV (Instructed - Uninstructed) | Positive effect | L Temporal Pole | -26 | 10 | -28 | 11 | 297 |
|  |  |  | L Cerebelum IV-V | -14 | -32 | -20 | 16 | 432 |
|  |  |  | Putamen L | -32 | 4 | -8 | 29 | 783 |
|  |  |  | L Mid Orbital Gyrus ( Area Fp2 ) | -2 | 50 | -4 | 67 | 1809 |
|  |  | Negative effect | L IFG p. Triangularis | -46 | 22 | 26 | 94 | 2538 |
|  |  |  | L Inferior Parietal Lobule ( Area PF (IPL)) | -56 | -38 | 46 | 11 | 297 |
|  |  |  | RPrecentral Gyrus ( Area 4p ) | 38 | -22 | 52 | 22 | 594 |

^d^ This table presents group results from voxelwise analyses of associations between expected value (based on fits to cue-evoked SCR) and brain activation on medium heat, as measured by AUC estimates (see Methods). Group results were analyzed using robust regression. See Methods for additional details.

Figure 6–Source Data 5. Comparing instructed and feedback-driven expected value (EV) within the Instructed Group^e^

| **Correction** | **Analysis** | **Effect** | **Anatomical label** | **x** | **y** | **z** | **# of voxels** | **Volume (mm^3^)** |
| --- | --- | --- | --- | --- | --- | --- | --- | --- |
| Pain modulatory network | Instructed vs Uninstructed EV | Positive association (Instructed > Uninstructed) | *No voxels survive* | | | | | |
|  |  | Negative association (Uninstructed > Instructed) | *No voxels survive* | | | | | |
|  | Instructed EV, controlling for uninstructed | Positive association with EV | L Putamen | -16 | 4 | -10 | 2 | 54 |
|  |  | Negative association with EV | *No voxels survive* | | | | | |
|  | Uninstructed EV, controlling for instructed | Positive association with EV | *No voxels survive* | | | | | |
|  |  | Negative association with EV | *No voxels survive* | | | | | |
| Whole brain correction | Instructed vs Uninstructed EV | Positive association (Instructed > Uninstructed) | R Middle Cingulate Cortex (Area 4a ) | 2 | -32 | 46 | 23 | 621 |
|  |  | Negative association (Uninstructed > Instructed) | *No voxels survive* | | | | | |
|  | Instructed EV, controlling for Uninstructed EV | Positive association with EV | L Insula Lobe | -28 | 8 | -16 | 2 | 54 |
|  |  |  | L Putamen / Nucleus Accumbens | -16 | 4 | -10 | 2 | 54 |
|  |  |  | R Middle Occipital Gyrus | 40 | -80 | 16 | 1 | 27 |
|  |  |  | R Caudate Nucleus | 14 | -8 | 20 | 4 | 108 |
|  |  |  | L MCC | -2 | -26 | 32 | 104 | 2808 |
|  |  |  | L rdACC | -16 | 22 | 26 | 5 | 135 |
|  |  |  | R Posterior Cingulate Cortex | 16 | -32 | 34 | 3 | 81 |
|  |  |  | R Superior Frontal Gyrus | 20 | 50 | 38 | 22 | 594 |
|  |  | Negative association with EV | *No voxels survive* | | | | | |
|  | Uninstructed EV, controlling for instructed | Positive association with EV | *No voxels survive* | | | | | |
|  |  | Negative association with EV | *No voxels survive* | | | | | |
| Uncorrected | Instructed vs Uninstructed EV | Positive association (Instructed > Uninstructed) | R dACC | 10 | 10 | 32 | 11 | 297 |
|  |  |  | R Middle Frontal Gyrus | 26 | 34 | 40 | 33 | 891 |
|  |  |  | R MCC ( Area 4a ) | 2 | -32 | 46 | 39 | 1053 |
|  |  | Negative association (Uninstructed > Instructed) | R Fusiform Gyrus | 22 | -32 | -20 | 12 | 324 |
|  | Instructed EV, controlling for uninstructed | Positive association with EV | R Insula Lobe | 38 | 22 | -8 | 35 | 945 |
|  |  |  | Substantia Nigra | 8 | -14 | -8 | 11 | 297 |
|  |  |  | L Superior Medial Gyrus (rACC, MPFC) | -14 | 52 | 8 | 44 | 1188 |
|  |  |  | R Caudate Nucleus | 14 | -8 | 20 | 4 | 108 |
|  |  |  | L MCC | -2 | -26 | 32 | 153 | 4131 |
|  |  |  | R dACC | 10 | 10 | 28 | 36 | 972 |
|  |  |  | R Posterior Cingulate Cortex | 16 | -32 | 34 | 3 | 81 |
|  |  |  | R Superior Frontal Gyrus | 22 | 46 | 40 | 49 | 1323 |
|  |  |  | L PreSMA | -16 | -8 | 56 | 17 | 459 |
|  |  | Negative association with EV | Area hOc3v [V3v] | -8 | -92 | -20 | 11 | 297 |
|  | Uninstructed EV, controlling for instructed | Positive association with EV | R Cerebelum IV-V | 22 | -32 | -22 | 11 | 297 |
|  |  | Negative association with EV | L Cerebelum VIII | -32 | -46 | -46 | 7 | 189 |
|  |  |  | R Cerebelum Crus 1 | 26 | -82 | -34 | 11 | 297 |
|  |  |  | L DLPFC | -32 | 20 | 26 | 21 | 567 |
|  |  |  | R Middle Frontal Gyrus | 26 | 32 | 40 | 14 | 378 |

^e^. This table presents group results from voxelwise analyses of associations between expected value and brain activation on medium heat within the Instructed Group (see Methods). Group results were analyzed using robust regression. Top rows are FDR-corrected within pain modulatory networks (see Figure 5 – Figure Supplement 1), middle rows are whole-brain corrected, and bottom rows are uncorrected at voxelwise p < .001. See Methods for additional details.

Figure 6–Source Data 6. Associations with unsigned prediction error (PE)^f^

| **Correction** | **Analysis** | **Effect** | **Anatomical label** | **x** | **y** | **z** | **# of voxels** | **Volume (mm^3^)** |
| --- | --- | --- | --- | --- | --- | --- | --- | --- |
| Pain modulatory network | Instructed Group | Positive association with PE | *No voxels survive* | | | | | |
|  |  | Negative association with PE | *No voxels survive* | | | | | |
|  | Uninstructed Group | Positive association with PE | *No voxels survive* | | | | | |
|  |  | Negative association with PE | *No voxels survive* | | | | | |
|  | Main effect of PE, controlling for Group | Positive effect | *No voxels survive* | | | | | |
|  |  | Negative effect | *No voxels survive* | | | | | |
|  | Group differences in PE (Instructed - Uninstructed) | Positive effect | *No voxels survive* | | | | | |
|  |  | Negative effect | *No voxels survive* | | | | | |
| Whole brain correction | Instructed Group | Positive association with PE | R Pallidum, contiguous with R Caudate, Ventral Striatum, Amygdala | 16 | 10 | -2 | 135 | 3645 |
|  |  |  | R DMPFC | 26 | 44 | 50 | 1 | 27 |
|  |  | Negative association with PE | R Superior Temporal Gyrus ( Area PFcm (IPL)) | 64 | -28 | 16 | 14 | 378 |
|  | Uninstructed Group | Positive association with PE | L Insula Lobe | -34 | 4 | 10 | 16 | 432 |
|  |  | Negative association with PE | *No voxels survive* | | | | | |
|  | Main effect of PE, controlling for Group | Positive effect | R Putamen, continguous with R caudate, R amygdala, R anterior insula | 20 | 10 | 2 | 343 | 9261 |
|  |  | Negative effect | R Superior Temporal Gyrus ( Area PF (IPL)), contiguous with R SII and R TPJ | 62 | -38 | 14 | 85 | 2295 |
|  | Group differences in PE (Instructed - Uninstructed) | Positive effect | *No voxels survive* | | | | | |
|  |  | Negative effect | R Superior Temporal Gyrus ( Area PF (IPL)), contiguous with R SII and R TPJ | 62 | -32 | 14 | 133 | 3591 |
| Uncorrected | Instructed Group | Positive association with PE | R Fusiform Gyrus | 38 | -16 | -38 | 21 | 567 |
|  |  |  | L Cerebelum Crus 2 | -28 | -88 | -40 | 4 | 108 |
|  |  |  | R Inferior Temporal Gyrus | 58 | -10 | -32 | 31 | 837 |
|  |  |  | L Pons | -4 | -16 | -22 | 50 | 1350 |
|  |  |  | R Caudate Nucleus, contiguous with putamen, amygdala, ventral striatum | 16 | 16 | -2 | 277 | 7479 |
|  |  |  | L Superior Frontal Gyrus ( Area Fp1 ) | -22 | 56 | 4 | 112 | 3024 |
|  |  |  | L Caudate Nucleus | -16 | 14 | 8 | 120 | 3240 |
|  |  |  | L IFG p. Triangularis ( Area 45 ) | -52 | 22 | 16 | 40 | 1080 |
|  |  |  | L IFG p. Triangularis | -40 | 44 | 10 | 96 | 2592 |
|  |  |  | L Superior Medial Gyrus | -10 | 34 | 34 | 27 | 729 |
|  |  | Negative association with PE | L Fusiform Gyrus ( Area FG3 ) | -34 | -50 | -16 | 132 | 3564 |
|  |  |  | L Fusiform Gyrus ( Area FG3 ) | -32 | -34 | -26 | 12 | 324 |
|  |  |  | R Middle Temporal Gyrus | 46 | -56 | 8 | 533 | 14391 |
|  |  |  | L Middle Occipital Gyrus ( Area hOc4la) | -38 | -80 | 14 | 193 | 5211 |
|  |  |  | L Superior Temporal Gyrus | -52 | -46 | 20 | 220 | 5940 |
|  |  |  | L Postcentral Gyrus ( Area 4p ) | -46 | -10 | 32 | 105 | 2835 |
|  |  |  | R Postcentral Gyrus ( Area 4p ) | 46 | -10 | 32 | 17 | 459 |
|  |  |  | L MCC | -10 | -16 | 44 | 41 | 1107 |
|  |  |  | RPrecentral Gyrus | 40 | -14 | 46 | 73 | 1971 |
|  |  |  | R Posterior-Medial Frontal | 10 | -2 | 74 | 24 | 648 |
|  | Uninstructed Group | Positive association with PE | R Fusiform Gyrus | 44 | -20 | -28 | 14 | 378 |
|  |  |  | R Calcarine Gyrus ( Area hOc1 [V1]) | 14 | -86 | -2 | 16 | 432 |
|  |  |  | L Insula Lobe, contiguous with putamen | -32 | 4 | 10 | 29 | 783 |
|  |  |  | RPrecentral Gyrus | -38 | -50 | 26 | 10 | 270 |
|  |  |  | L IFG p. Opercularis ( Area 44 ) | 52 | 2 | 38 | 18 | 486 |
|  |  | Negative association with PE | L Precentral Gyrus | -56 | 8 | 22 | 9 | 243 |
|  | Main effect of PE, controlling for Group | Positive effect | R Cerebelum Crus 2 | 46 | -64 | -44 | 15 | 405 |
|  |  |  | R Inferior Temporal Gyrus | 58 | -10 | -28 | 45 | 1215 |
|  |  |  | R Cerebelum Crus 1 | 32 | -70 | -28 | 31 | 837 |
|  |  |  | Brainstem (Pons) | -4 | -16 | -20 | 92 | 2484 |
|  |  |  | R Putamen, contiguous with R Caudate, anterior insula, amygdala | 20 | 10 | 2 | 483 | 13041 |
|  |  |  | R Rectal Gyrus | 14 | 28 | -20 | 17 | 459 |
|  |  |  | L Middle Temporal Gyrus | -52 | -40 | -10 | 13 | 351 |
|  |  |  | L Putamen, contiguous with Caudate, Anterior Insula | -20 | 10 | 8 | 166 | 4482 |
|  |  |  | L Middle Frontal Gyrus | -38 | 44 | 14 | 116 | 3132 |
|  |  |  | R Middle Frontal Gyrus | 32 | 46 | 32 | 60 | 1620 |
|  |  |  | R MCC | 4 | 28 | 34 | 89 | 2403 |
|  |  |  | R Middle Frontal Gyrus | 32 | 16 | 40 | 17 | 459 |
|  |  |  | R Superior Medial Gyrus | 8 | 28 | 62 | 34 | 918 |
|  |  | Negative effect | L Fusiform Gyrus ( Area FG3 ) | -32 | -50 | -14 | 102 | 2754 |
|  |  |  | R Inferior Temporal Gyrus | 44 | -58 | -10 | 21 | 567 |
|  |  |  | R Superior Temporal Gyrus | 58 | -38 | 14 | 143 | 3861 |
|  |  |  | L Superior Temporal Gyrus | -56 | -46 | 20 | 161 | 4347 |
|  |  |  | R Middle Occipital Gyrus | 32 | -74 | 20 | 125 | 3375 |
|  |  |  | L Postcentral Gyrus ( Area 4p ) | -44 | -10 | 32 | 65 | 1755 |
|  | Group differences in PE (Instructed - Uninstructed) | Positive effect | L Caudate Nucleus | -16 | 20 | 8 | 23 | 621 |
|  |  |  | R Anterior Insula | 28 | 28 | 10 | 17 | 459 |
|  |  |  | L IFG p. Triangularis ( Area 45 ) | -52 | 26 | 16 | 16 | 432 |
|  |  |  | L Superior Medial Gyrus | -8 | 34 | 38 | 21 | 567 |
|  |  | Negative effect | L Fusiform Gyrus ( Area FG4 ) | -38 | -50 | -22 | 94 | 2538 |
|  |  |  | R Cerebelum VI | 40 | -50 | -26 | 38 | 1026 |
|  |  |  | R Superior Temporal Gyrus | 52 | -46 | 14 | 399 | 10773 |
|  |  |  | L Middle Occipital Gyrus ( Area hOc4la) | -40 | -80 | 14 | 63 | 1701 |
|  |  |  | L Superior Temporal Gyrus | -52 | -44 | 16 | 180 | 4860 |
|  |  |  | R Cuneus | 16 | -70 | 26 | 38 | 1026 |
|  |  |  | L Postcentral Gyrus | -50 | -10 | 38 | 234 | 6318 |
|  |  |  | RPrecentral Gyrus | 44 | -10 | 44 | 186 | 5022 |
|  |  |  | L MCC ( Area 5M (SPL)) | -8 | -38 | 50 | 25 | 675 |
|  |  |  | R Posterior-Medial Frontal | 4 | -10 | 68 | 130 | 3510 |
|  |  |  | L Postcentral Gyrus ( Area 3b ) | -40 | -32 | 56 | 3 | 81 |

^f^. This table presents group results from voxelwise analyses of associations between unsigned prediction error (based on fits to pain) and brain activation on medium heat, as measured by AUC estimates (see Methods). Group results were analyzed using robust regression. See Methods for additional details.
