# Supplementary material for: Instructions and experiential learning have similar impacts on pain and pain-related brain responses but produce dissociations in value-based reversal learning
Source: eLife. 2022 Nov 1;11:e73353. doi: 10.7554/eLife.73353 (PMC9681218; doi:10.7554/eLife.73353)
Supplement: Figure 8—source data 2. [file elife-73353-fig8-data2.docx]

Figure 8–Source Data 2. Associations with expected value (EV): Uncorrected results.^b^

| **Analysis** | **Effect** | **Anatomical label** | **x** | **y** | **z** | **# of voxels** | **Volume (mm^3^)** |
| --- | --- | --- | --- | --- | --- | --- | --- |
| Instructed Group | Positive association with EV | L Entorhinal cortex, contiguous with L Putamen | -32 | 2 | -8 | 36 | 972 |
|  |  | L posterior Putamen | -32 | -10 | 8 | 8 | 216 |
|  |  | R ACC / MPFC | 14 | 40 | 14 | 5 | 135 |
|  |  | R Superior Frontal Gyrus (DMPFC) | 16 | 56 | 16 | 9 | 243 |
|  |  | rdACC (area 33) | 8 | 16 | 22 | 52 | 1404 |
|  |  | L rdACC | -14 | 28 | 26 | 16 | 432 |
|  |  | Posterior Cingulate Cortex | 2 | -38 | 26 | 31 | 837 |
|  |  | L M1, contiguous with S1, posterior cingulate cortex, precuneus | -20 | -22 | 40 | 207 | 5589 |
|  | Negative association with EV | L Superior Orbital Gyrus ( Area Fo3 ) | -22 | 44 | -14 | 22 | 594 |
|  |  | R Calcarine Gyrus ( Area hOc1 [V1]) | 16 | -56 | 8 | 40 | 1080 |
|  |  | L IFG p. Triangularis (DLPFC) | -46 | 28 | 22 | 65 | 1755 |
| Uninstructed Group | Positive association with EV | *No voxels survive* | | | | | |
|  | Negative association with EV | L Temporal Pole | -40 | 8 | -20 | 48 | 1296 |
|  |  | L IFG p. Orbitalis | -34 | 34 | -16 | 16 | 432 |
|  |  | R Hippocampus (DG) | 28 | -34 | -4 | 58 | 1566 |
|  |  | L Hippocampus | -34 | -38 | -4 | 20 | 540 |
|  |  | L Superior Orbital Gyrus ( Area Fp1 ) | -20 | 62 | -2 | 31 | 837 |
|  |  | L PCC | -2 | -46 | 14 | 161 | 4347 |
|  |  | L Caudate Tail, contiguous with L Thalamus, L Middle Insula | -26 | -22 | 20 | 71 | 1917 |
|  |  | L Insula Lobe | -26 | 26 | 10 | 12 | 324 |
| Main effect of EV, controlling for Group | Positive association with EV | R Cerebelum VIII | 26 | -68 | -56 | 27 | 729 |
|  |  | R Inferior Occipital Gyrus ( Area hOc3v [V3v]) | 34 | -92 | -10 | 21 | 567 |
|  |  | R SupraMarginal Gyrus ( Area PFm (IPL)) | 58 | -44 | 28 | 35 | 945 |
|  |  | L Middle Cingulate Cortex | -8 | -20 | 40 | 31 | 837 |
|  |  | L Postcentral Gyrus ( Area 3a ) | -32 | -34 | 46 | 10 | 270 |
|  |  | L Precentral Gyrus | -34 | -10 | 50 | 49 | 1323 |
|  |  | L Posterior-Medial Frontal | -10 | -8 | 58 | 43 | 1161 |
|  | Negative association with EV | L Temporal Pole | -40 | 10 | -22 | 34 | 918 |
|  |  | L Superior Orbital Gyrus ( Area Fo3 ) | -14 | 50 | -20 | 23 | 621 |
|  |  | R Temporal Pole | 38 | 8 | -22 | 11 | 297 |
|  |  | L Rectal Gyrus ( Area Fo2 ) | -8 | 20 | -22 | 16 | 432 |
|  |  | R Fusiform Gyrus | 38 | -26 | -20 | 4 | 108 |
|  |  | R Hippocampus (DG) | 26 | -32 | -8 | 96 | 2592 |
|  |  | L IFG p. Orbitalis | -34 | 40 | -16 | 37 | 999 |
|  |  | L Superior Orbital Gyrus (Fp1) | -28 | 62 | -2 | 31 | 837 |
|  |  | L Calcarine Gyrus | -8 | -50 | 4 | 46 | 1242 |
|  |  | R Calcarine Gyrus | 14 | -50 | 10 | 122 | 3294 |
|  |  | Thal: Temporal | -20 | -34 | 4 | 9 | 243 |
|  |  | Thal: Parietal | -20 | -22 | 16 | 34 | 918 |
| Group differences in EV (Instructed - Uninstructed) | Positive effect | R Cerebelum Crus 2 | 44 | -50 | -46 | 13 | 351 |
|  |  | L Anterior Insula / Operculum | -38 | 10 | -16 | 85 | 2295 |
|  |  | R Cerebelum VI | 40 | -62 | -22 | 9 | 243 |
|  |  | L MPFC, contiguous with rACC | -4 | 44 | 8 | 523 | 14121 |
|  |  | R Hippocampus | 32 | -32 | -4 | 32 | 864 |
|  |  | R Occipital cortex | 26 | -86 | 2 | 41 | 1107 |
|  |  | L Superior Temporal Gyrus | -56 | -14 | -2 | 9 | 243 |
|  |  | L Thalamus (Parietal) | -16 | -22 | 14 | 123 | 3321 |
|  |  | L Insula Lobe ( Area Ig2 ) | -38 | -16 | 10 | 35 | 945 |
|  |  | L PCC | -2 | -46 | 20 | 196 | 5292 |
|  | Negative effect | R Inferior Temporal Gyrus | 62 | -40 | -16 | 17 | 459 |
|  |  | L Inferior Temporal Gyrus | -64 | -46 | -14 | 13 | 351 |

^b^. This table presents group results from voxelwise analyses of associations between expected value (based on fits to pain) and brain activation on medium heat, as measured by AUC estimates (see Methods). Group results were analyzed using robust regression. All clusters are identified at a voxel-wise p-value of p < .001 (3 voxels at lowest threshold), contiguous with voxels at .005 and .01. See Methods for additional details.
