# Supplementary material for: Instructions and experiential learning have similar impacts on pain and pain-related brain responses but produce dissociations in value-based reversal learning
Source: eLife. 2022 Nov 1;11:e73353. doi: 10.7554/eLife.73353 (PMC9681218; doi:10.7554/eLife.73353)
Supplement: Figure 8—source data 3. [file elife-73353-fig8-data3.docx]

Figure 8–Source Data 3. Associations with expected value (EV) based on fits to heat-evoked SCR^c^

| **Correction** | **Analysis** | **Effect** | **Anatomical label** | **x** | **y** | **z** | **# of voxels** | **Volume (mm^3^)** |
| --- | --- | --- | --- | --- | --- | --- | --- | --- |
| Pain modulatory network | Instructed Group | Positive association with EV | L Putamen | -16 | 16 | -10 | 1 | 27 |
|  |  |  | R Insula Lobe | 32 | 20 | -10 | 8 | 216 |
|  |  |  | L sgACC | -10 | 34 | -4 | 5 | 135 |
|  |  |  | L rACC | 2 | 34 | 10 | 5 | 135 |
|  |  |  | R rACC | 14 | 44 | 14 | 4 | 108 |
|  |  | Negative association with EV | *No voxels survive* | | | | | |
|  | Uninstructed Group | Positive association with EV | *No voxels survive* | | | | | |
|  |  | Negative association with EV | *No voxels survive* | | | | | |
|  | Main effect of EV, controlling for Group | Positive effect | *No voxels survive* | | | | | |
|  |  | Negative effect | *No voxels survive* | | | | | |
|  | Group differences in EV (Instructed - Uninstructed) | Positive effect | *No voxels survive* | | | | | |
|  |  | Negative effect | *No voxels survive* | | | | | |
| Whole brain correction | Instructed Group | Positive association with EV | R Anterior Insula | 32 | 20 | -10 | 9 | 243 |
|  |  | Negative association with EV | *No voxels survive* | | | | | |
|  | Uninstructed Group | Positive association with EV | R DLPFC | 44 | 8 | 26 | 1 | 27 |
|  |  | Negative association with EV | L Anterior Insula | -26 | 26 | 10 | 12 | 324 |
|  |  |  | L rdACC | -16 | 40 | 16 | 2 | 54 |
|  | Main effect of EV, controlling for Group | Positive effect | *No voxels survive* | | | | | |
|  |  | Negative effect | *No voxels survive* | | | | | |
|  | Group differences in EV (Instructed - Uninstructed) | Positive effect | L Superior Medial Gyrus | -10 | 40 | 26 | 8 | 216 |
|  |  | Negative effect | *No voxels survive* | | | | | |
| Uncorrected | Instructed Group | Positive association with EV | L Putamen | -32 | 2 | -8 | 52 | 1404 |
|  |  |  | R Anterior Insula | 32 | 20 | -8 | 17 | 459 |
|  |  |  | R Caudate Nucleus | 16 | 20 | 4 | 33 | 891 |
|  |  |  | R rdACC | 14 | 40 | 14 | 7 | 189 |
|  |  |  | R rdACC | 8 | 16 | 22 | 29 | 783 |
|  |  |  | Middle Cingulate Cortex | 2 | -22 | 34 | 49 | 1323 |
|  |  | Negative association with EV | L OFC | -26 | 44 | -14 | 17 | 459 |
|  |  |  | R Retrosplenial Cortex | 20 | -52 | 8 | 14 | 378 |
|  |  |  | R Occipital Cortex | 8 | -64 | 14 | 23 | 621 |
|  |  |  | L DLPFC | -44 | 26 | 22 | 60 | 1620 |
|  | Uninstructed Group | Positive association with EV | R DLPFC | 58 | 16 | 16 | 16 | 432 |
|  |  | Negative association with EV | L Inferior Frontal Gyrus | -40 | 8 | -20 | 56 | 1512 |
|  |  |  | R Fusiform Gyrus | 28 | -34 | -4 | 61 | 1647 |
|  |  |  | L Fusiform Gyrus | -34 | -38 | -4 | 29 | 783 |
|  |  |  | L Prefrontal cortex | -22 | 64 | -2 | 21 | 567 |
|  |  |  | L Posterior hippocampus | -14 | -34 | 4 | 23 | 621 |
|  |  |  | L Anterior Insula | -26 | 28 | 10 | 25 | 675 |
|  |  |  | R Retrosplenial Cortex | 14 | -56 | 20 | 40 | 1080 |
|  |  |  | L Thalamus | -20 | -22 | 16 | 24 | 648 |
|  |  |  | L DMPFC | -14 | 40 | 26 | 8 | 216 |
|  | Main effect of EV, controlling for Group | Positive effect | R Cerebelum VIII | 26 | -68 | -56 | 26 | 702 |
|  |  |  | L Inferior Temporal Gyrus | -62 | -34 | -26 | 7 | 189 |
|  |  |  | R Inferior Occipital Gyrus ( Area hOc3v [V3v]) | 34 | -92 | -8 | 26 | 702 |
|  |  |  | R Insula Lobe | 32 | 22 | -4 | 34 | 918 |
|  |  |  | R MCC | 2 | -20 | 38 | 19 | 513 |
|  |  |  | L Postcentral Gyrus ( Area 3a ) | -32 | -34 | 46 | 6 | 162 |
|  |  |  | L Precentral Gyrus | -34 | -10 | 52 | 59 | 1593 |
|  |  |  | L Posterior-Medial Frontal | -10 | -8 | 58 | 29 | 783 |
|  |  | Negative effect | R Cerebelum Crus 1 | 14 | -76 | -28 | 4 | 108 |
|  |  |  | L medial OFC (Area Fo3 ) | -10 | 50 | -26 | 11 | 297 |
|  |  |  | R ParaHippocampal Gyrus | 28 | -38 | -8 | 39 | 1053 |
|  |  |  | R Precuneus | 16 | -46 | 10 | 47 | 1269 |
|  | Group differences in EV (Instructed - Uninstructed) | Positive effect | L Anterior Insula, contiguous with L Putamen | -38 | 4 | -16 | 78 | 2106 |
|  |  |  | L MPFC, ACC | -4 | 46 | 4 | 396 | 10692 |
|  |  |  | R Occipital Cortex | 26 | -86 | 2 | 57 | 1539 |
|  |  |  | L Thalamus (prefrontal) | -14 | -16 | 16 | 26 | 702 |
|  |  | Negative effect | *No voxels survive* | | | | | |

^c^. This table presents group results from voxelwise analyses of associations between expected value (based on fits to heat-evoked SCR) and brain activation on medium heat, as measured by AUC estimates (see Methods). Group results were analyzed using robust regression. See Methods for additional details.
