# Supplementary material for: Instructions and experiential learning have similar impacts on pain and pain-related brain responses but produce dissociations in value-based reversal learning
Source: eLife. 2022 Nov 1;11:e73353. doi: 10.7554/eLife.73353 (PMC9681218; doi:10.7554/eLife.73353)
Supplement: Figure 8—source data 4. [file elife-73353-fig8-data4.docx]

Figure 8–Source Data 4. Associations with expected value (EV) based on fits to cue-evoked SCR^d^

| **Correction** | **Analysis** | **Effect** | **Anatomical label** | **x** | **y** | **z** | **# of voxels** | **Volume (mm^3^)** |
| --- | --- | --- | --- | --- | --- | --- | --- | --- |
| Pain modulatory network | Instructed Group | Positive association with EV | L midbrain (substantia nigra) | -8 | -22 | -10 | 4 | 108 |
|  |  |  | L Thalamus (Parietal) | -22 | -22 | 16 | 1 | 27 |
|  |  | Negative association with EV | L Superior Orbital Gyrus / Area Fo1 | -14 | 44 | -22 | 10 | 270 |
|  |  |  | L IFG p. Triangularis | -44 | 34 | 14 | 2 | 54 |
|  |  |  | L IFG p. Triangularis | -44 | 22 | 22 | 17 | 459 |
|  | Uninstructed Group | Positive association with EV | *No voxels survive* | | | | | |
|  |  | Negative association with EV | *No voxels survive* | | | | | |
|  | Main effect of EV, controlling for Group | Positive effect | L midbrain (substantia nigra) | -8 | -20 | -10 | 2 | 54 |
|  |  | Negative effect | L IFG p. Triangularis | -40 | 34 | 14 | 7 | 189 |
|  | Group differences in EV (Instructed - Uninstructed) | Positive effect | *No voxels survive* | | | | | |
|  |  | Negative effect | *No voxels survive* | | | | | |
| Whole brain correction | Instructed Group | Positive association with EV | L Midbrain (Substantia Nigra) | -8 | -22 | -10 | 4 | 108 |
|  |  |  | dACC | 4 | 16 | 16 | 12 | 324 |
|  |  |  | L PreSMA | -20 | -32 | 44 | 1 | 27 |
|  |  | Negative association with EV | L Superior Orbital Gyrus / Fo1 | -14 | 44 | -20 | 18 | 486 |
|  |  |  | L IFG p. Triangularis | -38 | 44 | 4 | 4 | 108 |
|  |  |  | L IFG p. Triangularis | -46 | 22 | 26 | 150 | 4050 |
|  | Uninstructed Group | Positive association with EV | RPrecentral Gyrus | 46 | 2 | 46 | 10 | 270 |
|  |  |  | RPrecentral Gyrus | 32 | -22 | 52 | 1 | 27 |
|  |  |  | R Superior Frontal Gyrus | 26 | -8 | 64 | 12 | 324 |
|  |  | Negative association with EV | *No voxels survive* | | | | | |
|  | Main effect of EV, controlling for Group | Positive effect | *No voxels survive* | | | | | |
|  |  | Negative effect | *No voxels survive* | | | | | |
|  | Group differences in EV (Instructed - Uninstructed) | Positive effect | *No voxels survive* | | | | | |
|  |  | Negative effect | *No voxels survive* | | | | | |
| Uncorrected | Instructed Group | Positive association with EV | L Putamen, contiguous with Amygdala | -32 | 2 | -8 | 20 | 540 |
|  |  |  | R Middle Occipital Gyrus / Area hOc4la | 40 | -76 | 4 | 51 | 1377 |
|  |  |  | L Superior Frontal Gyrus | -22 | 38 | 50 | 6 | 162 |
|  |  | Negative association with EV | L Superior Orbital Gyrus / Area Fo1 | -14 | 44 | -20 | 25 | 675 |
|  |  |  | L IFG p. Triangularis | -46 | 22 | 26 | 201 | 5427 |
|  | Uninstructed Group | Positive association with EV | L DMPFC | -16 | -8 | 58 | 54 | 1458 |
|  |  |  | RPrecentral Gyrus | 46 | 2 | 46 | 14 | 378 |
|  |  |  | RPrecentral Gyrus | 28 | -4 | 50 | 29 | 783 |
|  |  |  | R Superior Frontal Gyrus | 28 | -8 | 64 | 22 | 594 |
|  |  | Negative association with EV | L Temporal Pole, contiguous with Insula | -40 | 8 | -20 | 53 | 1431 |
|  |  |  | R Hippocampus (DG) | 32 | -34 | -8 | 51 | 1377 |
|  |  |  | L Hippocampus (DG) | -34 | -34 | -8 | 31 | 837 |
|  |  |  | L Middle Orbital Gyrus | -32 | 40 | -8 | 6 | 162 |
|  |  |  | L ACC, MPFC | -2 | 40 | 4 | 41 | 1107 |
|  |  |  | R Calcarine Gyrus | 10 | -58 | 16 | 8 | 216 |
|  |  |  | R Caudate Nucleus | 20 | -22 | 22 | 16 | 432 |
|  |  |  | L Superior Medial Gyrus | -14 | 40 | 26 | 7 | 189 |
|  | Main effect of EV, controlling for Group | Positive effect | L Middle Cingulate Cortex, M1 | -16 | -14 | 46 | 66 | 1782 |
|  |  |  | L Inferior Parietal Lobule ( Area 1 ) | -52 | -26 | 50 | 23 | 621 |
|  |  | Negative effect | R VLPFC | 28 | 40 | -8 | 6 | 162 |
|  |  |  | L IFG p. Triangularis | -46 | 28 | 22 | 138 | 3726 |
|  | Group differences in EV (Instructed - Uninstructed) | Positive effect | L Temporal Pole | -26 | 10 | -28 | 11 | 297 |
|  |  |  | L Cerebelum IV-V | -14 | -32 | -20 | 16 | 432 |
|  |  |  | Putamen L | -32 | 4 | -8 | 29 | 783 |
|  |  |  | L Mid Orbital Gyrus ( Area Fp2 ) | -2 | 50 | -4 | 67 | 1809 |
|  |  | Negative effect | L IFG p. Triangularis | -46 | 22 | 26 | 94 | 2538 |
|  |  |  | L Inferior Parietal Lobule ( Area PF (IPL)) | -56 | -38 | 46 | 11 | 297 |
|  |  |  | RPrecentral Gyrus ( Area 4p ) | 38 | -22 | 52 | 22 | 594 |

^d^ This table presents group results from voxelwise analyses of associations between expected value (based on fits to cue-evoked SCR) and brain activation on medium heat, as measured by AUC estimates (see Methods). Group results were analyzed using robust regression. See Methods for additional details.
