# Supplementary material for: Instructions and experiential learning have similar impacts on pain and pain-related brain responses but produce dissociations in value-based reversal learning
Source: eLife. 2022 Nov 1;11:e73353. doi: 10.7554/eLife.73353 (PMC9681218; doi:10.7554/eLife.73353)
Supplement: Figure 8—source data 5. [file elife-73353-fig8-data5.docx]

Figure 8–Source Data 5. Comparing instructed and feedback-driven expected value (EV) within the Instructed Group^e^

| **Correction** | **Analysis** | **Effect** | **Anatomical label** | **x** | **y** | **z** | **# of voxels** | **Volume (mm^3^)** |
| --- | --- | --- | --- | --- | --- | --- | --- | --- |
| Pain modulatory network | Instructed vs Uninstructed EV | Positive association (Instructed > Uninstructed) | *No voxels survive* | | | | | |
|  |  | Negative association (Uninstructed > Instructed) | *No voxels survive* | | | | | |
|  | Instructed EV, controlling for uninstructed | Positive association with EV | L Putamen | -16 | 4 | -10 | 2 | 54 |
|  |  | Negative association with EV | *No voxels survive* | | | | | |
|  | Uninstructed EV, controlling for instructed | Positive association with EV | *No voxels survive* | | | | | |
|  |  | Negative association with EV | *No voxels survive* | | | | | |
| Whole brain correction | Instructed vs Uninstructed EV | Positive association (Instructed > Uninstructed) | R Middle Cingulate Cortex (Area 4a ) | 2 | -32 | 46 | 23 | 621 |
|  |  | Negative association (Uninstructed > Instructed) | *No voxels survive* | | | | | |
|  | Instructed EV, controlling for Uninstructed EV | Positive association with EV | L Insula Lobe | -28 | 8 | -16 | 2 | 54 |
|  |  |  | L Putamen / Nucleus Accumbens | -16 | 4 | -10 | 2 | 54 |
|  |  |  | R Middle Occipital Gyrus | 40 | -80 | 16 | 1 | 27 |
|  |  |  | R Caudate Nucleus | 14 | -8 | 20 | 4 | 108 |
|  |  |  | L MCC | -2 | -26 | 32 | 104 | 2808 |
|  |  |  | L rdACC | -16 | 22 | 26 | 5 | 135 |
|  |  |  | R Posterior Cingulate Cortex | 16 | -32 | 34 | 3 | 81 |
|  |  |  | R Superior Frontal Gyrus | 20 | 50 | 38 | 22 | 594 |
|  |  | Negative association with EV | *No voxels survive* | | | | | |
|  | Uninstructed EV, controlling for instructed | Positive association with EV | *No voxels survive* | | | | | |
|  |  | Negative association with EV | *No voxels survive* | | | | | |
| Uncorrected | Instructed vs Uninstructed EV | Positive association (Instructed > Uninstructed) | R dACC | 10 | 10 | 32 | 11 | 297 |
|  |  |  | R Middle Frontal Gyrus | 26 | 34 | 40 | 33 | 891 |
|  |  |  | R MCC ( Area 4a ) | 2 | -32 | 46 | 39 | 1053 |
|  |  | Negative association (Uninstructed > Instructed) | R Fusiform Gyrus | 22 | -32 | -20 | 12 | 324 |
|  | Instructed EV, controlling for uninstructed | Positive association with EV | R Insula Lobe | 38 | 22 | -8 | 35 | 945 |
|  |  |  | Substantia Nigra | 8 | -14 | -8 | 11 | 297 |
|  |  |  | L Superior Medial Gyrus (rACC, MPFC) | -14 | 52 | 8 | 44 | 1188 |
|  |  |  | R Caudate Nucleus | 14 | -8 | 20 | 4 | 108 |
|  |  |  | L MCC | -2 | -26 | 32 | 153 | 4131 |
|  |  |  | R dACC | 10 | 10 | 28 | 36 | 972 |
|  |  |  | R Posterior Cingulate Cortex | 16 | -32 | 34 | 3 | 81 |
|  |  |  | R Superior Frontal Gyrus | 22 | 46 | 40 | 49 | 1323 |
|  |  |  | L PreSMA | -16 | -8 | 56 | 17 | 459 |
|  |  | Negative association with EV | Area hOc3v [V3v] | -8 | -92 | -20 | 11 | 297 |
|  | Uninstructed EV, controlling for instructed | Positive association with EV | R Cerebelum IV-V | 22 | -32 | -22 | 11 | 297 |
|  |  | Negative association with EV | L Cerebelum VIII | -32 | -46 | -46 | 7 | 189 |
|  |  |  | R Cerebelum Crus 1 | 26 | -82 | -34 | 11 | 297 |
|  |  |  | L DLPFC | -32 | 20 | 26 | 21 | 567 |
|  |  |  | R Middle Frontal Gyrus | 26 | 32 | 40 | 14 | 378 |

^e^. This table presents group results from voxelwise analyses of associations between expected value and brain activation on medium heat within the Instructed Group (see Methods). Group results were analyzed using robust regression. Top rows are FDR-corrected within pain modulatory networks (see Figure 5 – Figure Supplement 1), middle rows are whole-brain corrected, and bottom rows are uncorrected at voxelwise p < .001. See Methods for additional details.
