# Supplementary material for: Instructions and experiential learning have similar impacts on pain and pain-related brain responses but produce dissociations in value-based reversal learning
Source: eLife. 2022 Nov 1;11:e73353. doi: 10.7554/eLife.73353 (PMC9681218; doi:10.7554/eLife.73353)
Supplement: Figure 8—source data 6. [file elife-73353-fig8-data6.docx]

Figure 8–Source Data 6. Associations with unsigned prediction error (PE)^f^

| **Correction** | **Analysis** | **Effect** | **Anatomical label** | **x** | **y** | **z** | **# of voxels** | **Volume (mm^3^)** |
| --- | --- | --- | --- | --- | --- | --- | --- | --- |
| Pain modulatory network | Instructed Group | Positive association with PE | *No voxels survive* | | | | | |
|  |  | Negative association with PE | *No voxels survive* | | | | | |
|  | Uninstructed Group | Positive association with PE | *No voxels survive* | | | | | |
|  |  | Negative association with PE | *No voxels survive* | | | | | |
|  | Main effect of PE, controlling for Group | Positive effect | *No voxels survive* | | | | | |
|  |  | Negative effect | *No voxels survive* | | | | | |
|  | Group differences in PE (Instructed - Uninstructed) | Positive effect | *No voxels survive* | | | | | |
|  |  | Negative effect | *No voxels survive* | | | | | |
| Whole brain correction | Instructed Group | Positive association with PE | R Pallidum, contiguous with R Caudate, Ventral Striatum, Amygdala | 16 | 10 | -2 | 135 | 3645 |
|  |  |  | R DMPFC | 26 | 44 | 50 | 1 | 27 |
|  |  | Negative association with PE | R Superior Temporal Gyrus ( Area PFcm (IPL)) | 64 | -28 | 16 | 14 | 378 |
|  | Uninstructed Group | Positive association with PE | L Insula Lobe | -34 | 4 | 10 | 16 | 432 |
|  |  | Negative association with PE | *No voxels survive* | | | | | |
|  | Main effect of PE, controlling for Group | Positive effect | R Putamen, continguous with R caudate, R amygdala, R anterior insula | 20 | 10 | 2 | 343 | 9261 |
|  |  | Negative effect | R Superior Temporal Gyrus ( Area PF (IPL)), contiguous with R SII and R TPJ | 62 | -38 | 14 | 85 | 2295 |
|  | Group differences in PE (Instructed - Uninstructed) | Positive effect | *No voxels survive* | | | | | |
|  |  | Negative effect | R Superior Temporal Gyrus ( Area PF (IPL)), contiguous with R SII and R TPJ | 62 | -32 | 14 | 133 | 3591 |
| Uncorrected | Instructed Group | Positive association with PE | R Fusiform Gyrus | 38 | -16 | -38 | 21 | 567 |
|  |  |  | L Cerebelum Crus 2 | -28 | -88 | -40 | 4 | 108 |
|  |  |  | R Inferior Temporal Gyrus | 58 | -10 | -32 | 31 | 837 |
|  |  |  | L Pons | -4 | -16 | -22 | 50 | 1350 |
|  |  |  | R Caudate Nucleus, contiguous with putamen, amygdala, ventral striatum | 16 | 16 | -2 | 277 | 7479 |
|  |  |  | L Superior Frontal Gyrus ( Area Fp1 ) | -22 | 56 | 4 | 112 | 3024 |
|  |  |  | L Caudate Nucleus | -16 | 14 | 8 | 120 | 3240 |
|  |  |  | L IFG p. Triangularis ( Area 45 ) | -52 | 22 | 16 | 40 | 1080 |
|  |  |  | L IFG p. Triangularis | -40 | 44 | 10 | 96 | 2592 |
|  |  |  | L Superior Medial Gyrus | -10 | 34 | 34 | 27 | 729 |
|  |  | Negative association with PE | L Fusiform Gyrus ( Area FG3 ) | -34 | -50 | -16 | 132 | 3564 |
|  |  |  | L Fusiform Gyrus ( Area FG3 ) | -32 | -34 | -26 | 12 | 324 |
|  |  |  | R Middle Temporal Gyrus | 46 | -56 | 8 | 533 | 14391 |
|  |  |  | L Middle Occipital Gyrus ( Area hOc4la) | -38 | -80 | 14 | 193 | 5211 |
|  |  |  | L Superior Temporal Gyrus | -52 | -46 | 20 | 220 | 5940 |
|  |  |  | L Postcentral Gyrus ( Area 4p ) | -46 | -10 | 32 | 105 | 2835 |
|  |  |  | R Postcentral Gyrus ( Area 4p ) | 46 | -10 | 32 | 17 | 459 |
|  |  |  | L MCC | -10 | -16 | 44 | 41 | 1107 |
|  |  |  | RPrecentral Gyrus | 40 | -14 | 46 | 73 | 1971 |
|  |  |  | R Posterior-Medial Frontal | 10 | -2 | 74 | 24 | 648 |
|  | Uninstructed Group | Positive association with PE | R Fusiform Gyrus | 44 | -20 | -28 | 14 | 378 |
|  |  |  | R Calcarine Gyrus ( Area hOc1 [V1]) | 14 | -86 | -2 | 16 | 432 |
|  |  |  | L Insula Lobe, contiguous with putamen | -32 | 4 | 10 | 29 | 783 |
|  |  |  | RPrecentral Gyrus | -38 | -50 | 26 | 10 | 270 |
|  |  |  | L IFG p. Opercularis ( Area 44 ) | 52 | 2 | 38 | 18 | 486 |
|  |  | Negative association with PE | L Precentral Gyrus | -56 | 8 | 22 | 9 | 243 |
|  | Main effect of PE, controlling for Group | Positive effect | R Cerebelum Crus 2 | 46 | -64 | -44 | 15 | 405 |
|  |  |  | R Inferior Temporal Gyrus | 58 | -10 | -28 | 45 | 1215 |
|  |  |  | R Cerebelum Crus 1 | 32 | -70 | -28 | 31 | 837 |
|  |  |  | Brainstem (Pons) | -4 | -16 | -20 | 92 | 2484 |
|  |  |  | R Putamen, contiguous with R Caudate, anterior insula, amygdala | 20 | 10 | 2 | 483 | 13041 |
|  |  |  | R Rectal Gyrus | 14 | 28 | -20 | 17 | 459 |
|  |  |  | L Middle Temporal Gyrus | -52 | -40 | -10 | 13 | 351 |
|  |  |  | L Putamen, contiguous with Caudate, Anterior Insula | -20 | 10 | 8 | 166 | 4482 |
|  |  |  | L Middle Frontal Gyrus | -38 | 44 | 14 | 116 | 3132 |
|  |  |  | R Middle Frontal Gyrus | 32 | 46 | 32 | 60 | 1620 |
|  |  |  | R MCC | 4 | 28 | 34 | 89 | 2403 |
|  |  |  | R Middle Frontal Gyrus | 32 | 16 | 40 | 17 | 459 |
|  |  |  | R Superior Medial Gyrus | 8 | 28 | 62 | 34 | 918 |
|  |  | Negative effect | L Fusiform Gyrus ( Area FG3 ) | -32 | -50 | -14 | 102 | 2754 |
|  |  |  | R Inferior Temporal Gyrus | 44 | -58 | -10 | 21 | 567 |
|  |  |  | R Superior Temporal Gyrus | 58 | -38 | 14 | 143 | 3861 |
|  |  |  | L Superior Temporal Gyrus | -56 | -46 | 20 | 161 | 4347 |
|  |  |  | R Middle Occipital Gyrus | 32 | -74 | 20 | 125 | 3375 |
|  |  |  | L Postcentral Gyrus ( Area 4p ) | -44 | -10 | 32 | 65 | 1755 |
|  | Group differences in PE (Instructed - Uninstructed) | Positive effect | L Caudate Nucleus | -16 | 20 | 8 | 23 | 621 |
|  |  |  | R Anterior Insula | 28 | 28 | 10 | 17 | 459 |
|  |  |  | L IFG p. Triangularis ( Area 45 ) | -52 | 26 | 16 | 16 | 432 |
|  |  |  | L Superior Medial Gyrus | -8 | 34 | 38 | 21 | 567 |
|  |  | Negative effect | L Fusiform Gyrus ( Area FG4 ) | -38 | -50 | -22 | 94 | 2538 |
|  |  |  | R Cerebelum VI | 40 | -50 | -26 | 38 | 1026 |
|  |  |  | R Superior Temporal Gyrus | 52 | -46 | 14 | 399 | 10773 |
|  |  |  | L Middle Occipital Gyrus ( Area hOc4la) | -40 | -80 | 14 | 63 | 1701 |
|  |  |  | L Superior Temporal Gyrus | -52 | -44 | 16 | 180 | 4860 |
|  |  |  | R Cuneus | 16 | -70 | 26 | 38 | 1026 |
|  |  |  | L Postcentral Gyrus | -50 | -10 | 38 | 234 | 6318 |
|  |  |  | RPrecentral Gyrus | 44 | -10 | 44 | 186 | 5022 |
|  |  |  | L MCC ( Area 5M (SPL)) | -8 | -38 | 50 | 25 | 675 |
|  |  |  | R Posterior-Medial Frontal | 4 | -10 | 68 | 130 | 3510 |
|  |  |  | L Postcentral Gyrus ( Area 3b ) | -40 | -32 | 56 | 3 | 81 |

^f^. This table presents group results from voxelwise analyses of associations between unsigned prediction error (based on fits to pain) and brain activation on medium heat, as measured by AUC estimates (see Methods). Group results were analyzed using robust regression. See Methods for additional details.
