# Supplementary material for: Instructions and experiential learning have similar impacts on pain and pain-related brain responses but produce dissociations in value-based reversal learning
Source: eLife. 2022 Nov 1;11:e73353. doi: 10.7554/eLife.73353 (PMC9681218; doi:10.7554/eLife.73353)
Supplement: Supplementary file 1. [file elife-73353-supp1.docx]

Supplementary File 1A. Heat intensity effects on pain in fMRI participants (n = 36).^a^

^a^. This table presents results of a linear mixed model predicting subjective pain as a function of Heat Level (High vs Medium vs Low), Group (Instructed vs Uninstructed), Cue (Original High vs Original Low), and Phase (Original vs Reversed) in subjects with useable fMRI data (n = 36). Model specification is identical to Table 1. Trials that were omitted from fMRI analyses (e.g. due to head motion or scanner artifacts) were also omitted for consistency with neuroimaging analyses.

^b^. Estimates based on a linear mixed effects model implemented in the “lmer” function of lme4 (Bates et al., 2015) using the following code: lmer(Pain~group*templevels*cue*phase+(1+templevels+cue*phase||subject)).

^c^. Estimates based on a linear mixed effects model implemented in the “lme” function of nlme (Pinheiro et al., 2021) including autoregression using the following code: lme(Pain~group*templevels*cue*phase, random=~1+templevels+cue*phase|subject, correlation=corAR1(), na.action=na.exclude).

^d^. Estimates based on Bayesian model linear mixed models using the “brms” function (Bürkner, 2017) using the following code: brm(Pain~group*templevels*cue*phase+(1+templevels+cue*phase|subject,prior=set_prior("normal(0,2.5)", class="b"), save_all_pars=TRUE, silent=TRUE, refresh=0, iter = 4000, warmup = 1000). Posterior estimates and the Region of Partial Equivalence were obtained using the “describe_posterior” function from the package BayesTestR (Makowski et al., 2019a) and interpreted as in (Makowski et al., 2019b). The Region of Partial Equivalence (ROPE) was defined as [-0.24, 0.24].

Supplementary File 1B. Effects on medium heat pain within fMRI participants (n = 36).^a^

^ a^. This table presents results of a linear mixed model predicting subjective pain on medium heat trials as a function of Group (Instructed vs Uninstructed), Cue (Original High vs Original Low), and Phase (Original vs Reversed) across all fMRI participants (n = 36). See Table 1 for additional information about model specification and presentation.

^b^. Estimates based on a linear mixed effects model implemented in the “lmer” function of lme4 (Bates et al., 2015) using the following code: lmer(Pain_Medium_~Group*Cue*Phase+(1+ Cue*Phase|Subject)).

^c^. Estimates based on a linear mixed effects model implemented in the “lme” function of nlme (Pinheiro et al., 2021) including autoregression using the following code: lme(Pain~Group *Cue*Phase, random=~1+Cue*Phase|Subject, correlation=corAR1(), na.action=na.exclude).

^d^. Estimates based on Bayesian model linear mixed models using the “brms” function (Bürkner, 2017) using the following code: brm(Pain~Group *Cue*Phase+(1+Cue*Phase|Subject,prior=set_prior("normal(0,2.5)", class="b"), save_all_pars=TRUE, silent=TRUE, refresh=0, iter = 4000, warmup = 1000). Posterior estimates and the Region of Partial Equivalence were obtained using the “describe_posterior” function from the package BayesTestR (Makowski et al., 2019a) and interpreted as in (Makowski et al., 2019b). The Region of Partial Equivalence (ROPE) was defined as [-0.17, 0.17].
