# Supplementary material for: Instructions and experiential learning have similar impacts on pain and pain-related brain responses but produce dissociations in value-based reversal learning
Source: eLife. 2022 Nov 1;11:e73353. doi: 10.7554/eLife.73353 (PMC9681218; doi:10.7554/eLife.73353)
Supplement: Supplementary file 2. [file elife-73353-supp2.docx]

Supplementary File 2. Bayesian multilevel model evaluating effects of Group, Cue, Phase, and Trial on medium heat pain using Beta family.^a^

^a^. Estimates based on Bayesian model linear mixed models using the “brms” function (Bürkner, 2017) using the following code: brm(Pain~group*cue*phase+trial+(1+cue*phase|subject,prior=set_prior("normal(0,2.5)", class="b"), family = Beta(), save_all_pars=TRUE, silent=TRUE, refresh=0, iter = 4000, warmup = 1000). Posterior estimates and the Region of Partial Equivalence were obtained using the “describe_posterior” function from the package BayesTestR (Makowski et al., 2019a) and interpreted as in (Makowski et al., 2019b). The Region of Partial Equivalence (ROPE) was defined as [-0.236, 0.236].
